# Repression of EEF1D by KSHV RTA promotes viral lytic reactivation

**DOI:** 10.1128/jvi.01793-25

**Published:** 2026-03-10

**Authors:** Min Xiang, Lei Yu, Chunyan Han, Liang Huang, Lianghui Dong, Lei Bai, Shuwen Wu, Ke Lan

**Affiliations:** 1 State Key Laboratory of Virology and Biosafety, College of Life Sciences, Wuhan University 98436, Wuhan, China; 2 Department of Infectious Diseases, Frontier Science Center for Immunology and Metabolism, Medical Research Institute, Zhongnan Hospital of Wuhan University 619779 , Wuhan, China; 3 Taikang Center for Life and Medical Sciences, Wuhan University, Wuhan, China; University of Toronto, Toronto, Ontario, Canada

**Keywords:** KSHV, RTA, EEF1D, DNMT3A, PATZ1

## Abstract

Kaposi’s sarcoma-associated herpesvirus (KSHV) establishes lifelong latency in the host but can reactivate in the lytic cycle under specific pathophysiological conditions. The replication and transcription activator (RTA) serves as the master regulator of this latent-to-lytic switch. Although RTA is well known as a potent viral transcriptional activator and E3 ubiquitin ligase, its role in modulating host gene expression remains incompletely understood. In this study, we identified eukaryotic translation elongation factor 1δ (EEF1D) as a previously unrecognized host inhibitory factor that inhibits KSHV reactivation. Functional analyses revealed that ectopic expression of EEF1D suppressed viral lytic replication, whereas EEF1D depletion enhanced viral reactivation. KSHV RTA counteracts this inhibition through two complementary mechanisms: interaction with EEF1D protein to promote its ubiquitin-proteasome-mediated degradation and, more prominently, repression of *EEF1D* transcription through promoter silencing. Dual-luciferase reporter assays further revealed that this transcriptional repression is conserved among primate γ-herpesviruses, including KSHV, rhesus rhadinovirus, and Epstein-Barr virus, but is absent in murine γ-herpesvirus 68. Mechanistically, RTA induces DNMT3A-dependent hypermethylation of the *EEF1D* promoter, a process facilitated by the transcription factor, PATZ1. Collectively, these findings reveal a previously unrecognized repressive function of RTA on the host gene *EEF1D*, highlighting an additional layer of viral strategy to promote lytic reactivation.

**IMPORTANCE:**

Kaposi's sarcoma-associated herpesvirus (KSHV) establishes lifelong latency in host cells but can periodically reactivate, a process that is essential for viral spread and disease development. The viral replication and transcription activator (RTA) serves as the master switch of this transition; however, the host factors that inhibit reactivation and the mechanisms by which RTA overcomes them remain incompletely defined. In this study, we identified the host protein eukaryotic translation elongation factor 1δ (EEF1D) as a previously unrecognized inhibitor of KSHV lytic replication. However, RTA inhibited EEF1D expression at both the protein and transcriptional levels. These findings expand the functional repertoire of RTA by revealing its ability to repress host gene transcription, providing new insights into viral persistence and pathogenesis.

## INTRODUCTION

Kaposi’s sarcoma-associated herpesvirus (KSHV), also known as human herpesvirus 8, is a γ-herpesvirus associated with several human malignancies, including Kaposi’s sarcoma, primary effusion lymphoma (PEL), and multicentric Castleman’s disease (MCD) (1–4). Like other herpesviruses, KSHV alternates between two life cycle phases: latency and lytic replication (5). During latency, the viral episome is tethered to host chromosomes, enabling persistence with limited antigen expression and immune evasion (6–13). Various environmental and host factors, including hypoxia, oxidative stress, immune dysregulation, and inflammatory signals, can trigger reactivation into the lytic cycle (14–18). Lytic replication involves the temporally ordered expression of immediate-early, early, and late genes, leading to viral DNA amplification and the production of infectious virions (5). In addition to enabling viral spread, lytic reactivation contributes to oncogenesis by inducing host cytokines and growth factors that support the proliferation of latently infected cells (19, 20). Clinically, ganciclovir treatment not only decreases detectable KSHV DNA but also reduces the frequency of MCD flares (21), highlighting the pivotal role of reactivation in KSHV-driven tumorigenesis.

The KSHV replication and transcription activator (RTA), encoded by ORF50, is the master regulator of KSHV reactivation (22, 23). Multiple studies have shown that ectopic expression of RTA in PEL cells is sufficient to trigger a complete lytic program. This process involves the induction of a wide range of lytic genes, including vIL-6, PAN, ORF59, ORF65, and K8.1, and the production of encapsidated, DNase-resistant viral genomes, underscoring RTA’s central role in driving lytic reactivation (22–24). RTA functions through two well-established mechanisms. First, it acts as a potent transcriptional activator through directly binding viral promoters or cooperating with host factors such as RBP-Jκ, C/EBPα, and Oct-1 (25–32). Second, RTA exhibits E3 ubiquitin ligase activity, targeting host antiviral proteins, including IRF-7, STAT6, K-RBP, and SMC5/6, as well as viral proteins such as vFLIP, for proteasomal degradation (33–37). However, accumulating evidence indicates that RTA also represses the transcription of host genes, such as *MDM2*, *MyD88*, and *ID2*, through mechanisms not explained by its known activities (38–40). Interestingly, the Epstein-Barr virus homolog BRLF1 has been reported to downregulate *MYC*, *IRF3*, and *IRF7* (41, 42). This dual activity of the transcription factor resembles that of HSV-1 ICP4 and cellular transcriptional regulators, including Runx2, KAP1, and KDM5C, which can act as activators or repressors depending on the interacting partners (43–46). Despite these observations, the mechanisms underlying RTA-mediated transcriptional repression remain poorly defined.

The eukaryotic translation elongation factor 1δ (*EEF1D*) encodes a 647-amino-acid protein (the long isoform) generated via alternative splicing. Beyond its canonical role in translation, EEF1D functions as a transcriptional regulator of genes containing heat-shock elements (HSEs) (47). EEF1D has been shown to induce HSE-containing genes in cooperation with heat shock transcription factor 1 (HSF1) and NF-E2-related factor 2 (Nrf2) (47). Heat shock proteins (HSPs), driven by HSEs, act as ubiquitous molecular chaperones essential for protein folding and proteostasis under both physiological and stress conditions (48). Recent evidence indicates that the long coding RNA NONMMUT033452.2-EEF1D-HSP regulatory axis is critical for maintaining respiratory epithelial integrity and is strongly associated with asthma pathogenesis (49). In addition to stress responses, EEF1D participates in diverse cellular processes, including cell proliferation and signal transduction (47, 50). For instance, it promotes glioma cell proliferation, migration, and invasion through epithelial-mesenchymal transition and activation of the PI3K/Akt pathway (50). Moreover, heat shock proteins downstream of EEF1D mitigate reactive oxygen species (ROS) accumulation, providing cytoprotective effects. EEF1D dysregulation has been implicated in tumorigenesis and neurodegenerative disorders (51, 52), highlighting its multifaceted biological functions. Despite these insights, the role of EEF1D in viral infection remains largely unexplored.

In our previous study, we identified EEF1D as a candidate binding partner of RTA (53). In this study, we confirmed the interaction between RTA and EEF1D and demonstrated that EEF1D acts as a previously unrecognized host inhibition factor, as its overexpression suppresses viral lytic reactivation, whereas its depletion facilitates it. We further showed that RTA overcomes this inhibition through two coordinated mechanisms: promoting proteasomal degradation of EEF1D via its E3 ubiquitin ligase activity and, more prominently, by repressing *EEF1D* transcription. Reporter assays revealed that this transcriptional repression is conserved among KSHV, rhesus rhadinovirus (RRV), and Epstein-Barr virus (EBV), but is absent in murine γ-herpesvirus 68 (MHV68). In addition, we found that RTA induces DNMT3A-dependent hypermethylation of the *EEF1D* promoter through PATZ1-mediated recruitment. Together, these results establish a previously unrecognized role of RTA as a transcriptional repressor of a host gene, highlighting a new strategy by which KSHV promotes lytic replication.

## RESULTS

### EEF1D interacts with KSHV RTA

In our previous study, we identified EEF1D as a putative binding partner of KSHV RTA using mass spectrometry (53). Given that EEF1D exists as both a canonical short isoform and a long isoform (54), we first sought to determine which isoform mediates the interaction. Co-immunoprecipitation (Co-IP) assays demonstrated that RTA specifically interacts with the long isoform (647 aa, ~95 kDa) but not the short isoform (Fig. S1). Consequently, we refer to this long isoform simply as EEF1D throughout the text.

**Fig 1:**
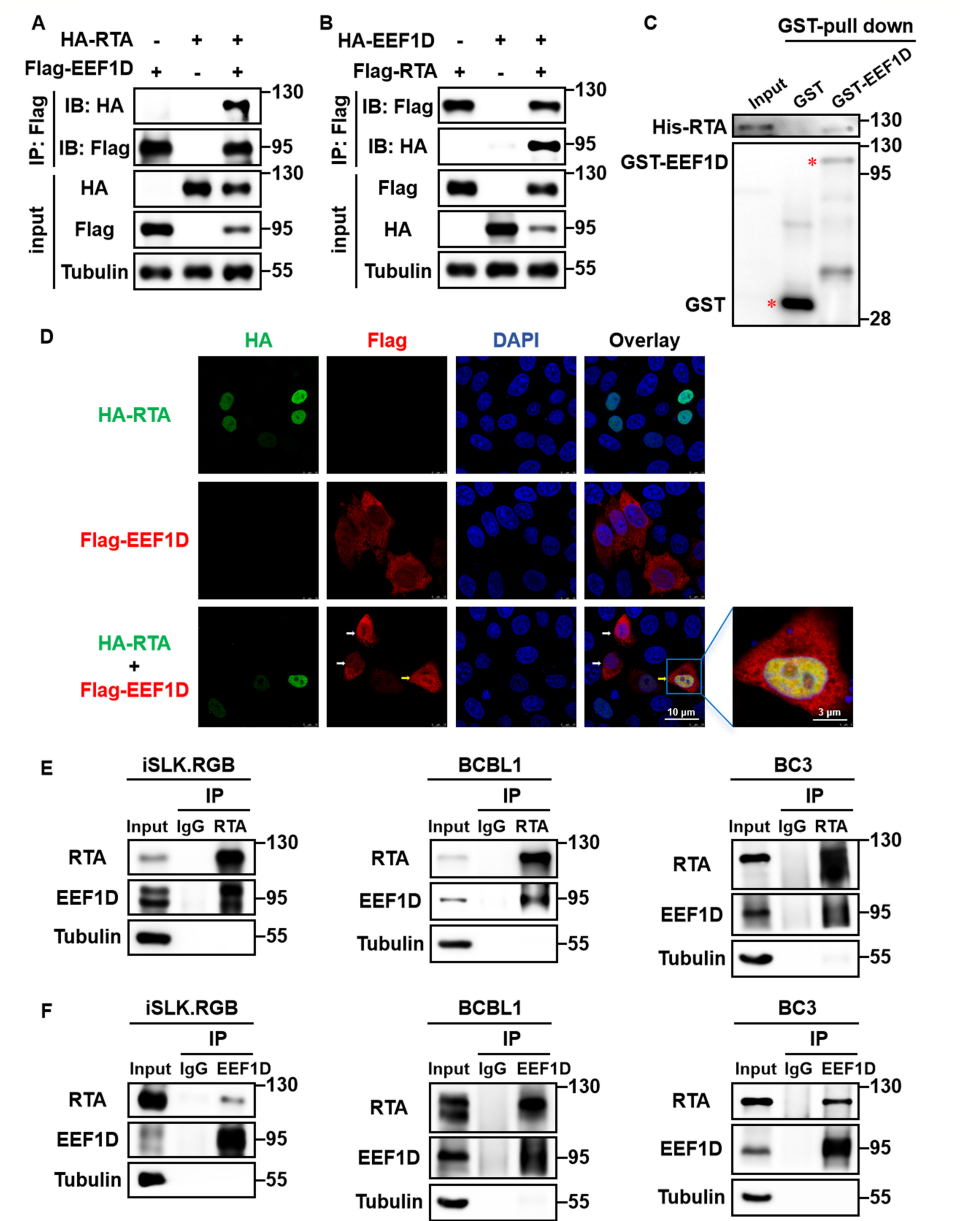
EEF1D interacts with KSHV RTA. (**A and B**) Co-IP in HEK293T cells. Cells were transfected with the indicated plasmids and harvested 48 h after transfection. Lysates were immunoprecipitated with anti-Flag M2 affinity gel and analyzed by immunoblotting using the indicated antibodies. (**C**) *In vitro* GST pull-down assay. Bacterially expressed GST-EEF1D or GST bound to glutathione-magnetic beads was incubated with purified His-RTA, and the bound proteins were analyzed by immunoblotting. (**D**) Confocal microscopy of HeLa cells. The cells were transfected with the indicated plasmids. When cell confluence reached approximately 90%, cells were fixed, permeabilized, and stained with mouse anti-Flag and rabbit anti-HA antibodies, followed by Alexa Fluor 488-goat anti-rabbit IgG (green), Alexa Fluor 555-goat anti-mouse IgG (red), and DAPI (blue). Images were acquired using a Leica SP8 confocal microscope. In the bottom row, white arrows indicate Flag-EEF1D single-positive cells, and the yellow arrow indicates a cell co-expressing HA-RTA and Flag-EEF1D in the same field of view. (**E**) Co-IP of endogenous proteins in KSHV-infected iSLK.RGB, BCBL1, and BC3 cells. Lytic reactivation was induced with Dox (2 μg/mL) or TPA (20 ng/mL) for 48 h. Lysates were immunoprecipitated with an anti-RTA antibody or rabbit IgG control and analyzed by immunoblotting. (**F**) Reciprocal Co-IP in the same cells as in panel E. Lysates were immunoprecipitated with an anti-EEF1D antibody or rabbit IgG control and subsequently analyzed by immunoblotting.

To further validate this interaction, HEK293T cells were co-transfected with HA-RTA and Flag-EEF1D. Co-IP assays confirmed their specific association, which was further substantiated by reciprocal Co-IP assays (Fig. 1A and B). To determine whether this interaction was direct, an *in vitro* GST pull-down assay was performed. Purified His-RTA was incubated with GST-EEF1D or a GST control immobilized on glutathione-magnetic beads. Immunoblot analysis revealed a direct interaction between RTA and EEF1D (Fig. 1C).

To complement these biochemical findings, immunofluorescence assays were performed to assess the subcellular localizations of EEF1D and RTA. HeLa cells transiently co-expressing Flag-EEF1D and HA-RTA exhibited clear nuclear colocalization, as visualized by confocal microscopy (Fig. 1D). Notably, a direct comparison of cells within the same field (Fig. 1D, bottom row) revealed that whereas EEF1D exhibited a predominantly cytoplasmic distribution when expressed alone, it co-localized with RTA in the nucleus upon co-expression, demonstrating the RTA-dependent nuclear recruitment of EEF1D.

Next, we assessed whether RTA and EEF1D interact endogenously. RTA expression was induced to detectable levels via doxycycline (Dox) treatment in iSLK.RGB cells carrying a Dox-inducible RTA cassette (55), and via 12-O-tetradecanoylphorbol-13-acetate (TPA) treatment in BCBL1 and BC3 cells. Immunoblotting of whole-cell lysates (input) confirmed the endogenous expression of EEF1D in these cell lines (Fig. 1E). Under these conditions, endogenous EEF1D co-precipitated with RTA in all three cell systems (Fig. 1E), and reciprocal Co-IP assays confirmed this interaction (Fig. 1F). Collectively, these findings identify EEF1D as a novel cellular protein that directly interacts with KSHV RTA.

### Ectopic expression of EEF1D suppresses KSHV lytic replication

**Fig 2:**
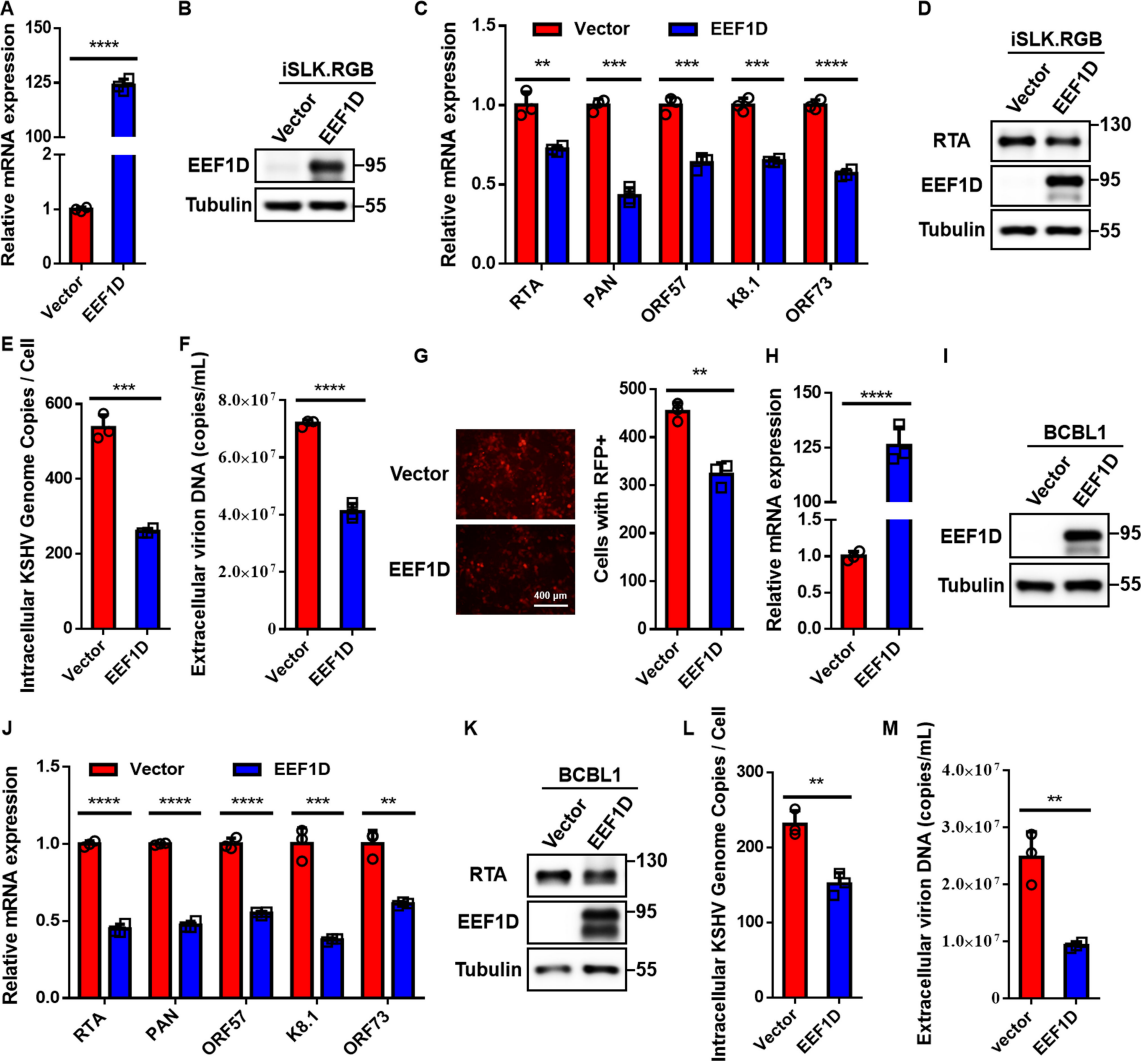
Ectopic expression of EEF1D suppresses KSHV lytic replication. (**A and B**) iSLK.RGB cells were stably transduced with lentiviral vectors expressing Flag-EEF1D or an empty vector to generate iSLK.RGB-Flag-EEF1D and iSLK.RGB-Flag-vector cell lines. EEF1D overexpression was confirmed by qRT-PCR (**A**) and immunoblotting with a specific anti-EEF1D antibody (**B**). (**C**) Stable EEF1D-overexpressing and vector control cells were treated with Dox to induce KSHV reactivation. At 72 h post-induction, the transcript levels of representative lytic genes (*RTA*, *PAN*, *ORF57*, *and K8.1*) and the latent gene *ORF73* were quantified using qRT-PCR. (**D**) Protein levels of RTA, EEF1D, and Tubulin in lysates from cells treated identically to those in (**C**) were analyzed by immunoblotting. (**E and F**) Intracellular (**E**) and extracellular (**F**) KSHV DNA copy numbers in the same cells as in panel C were quantified using qPCR. Intracellular viral load was expressed as copies per cell, and extracellular viral load was expressed as copies per mL. (**G**) Culture supernatants (2 mL) collected 96 h post-induction were used to infect HEK293T cells. At 24 h post-infection, RFP expression was visualized using fluorescence microscopy. Images were segmented using Cellpose, and the number of RFP-positive cells was quantified using the ImageJ software. (**H and I**) BCBL1 cells were stably transduced to express Flag-EEF1D or vector control. EEF1D overexpression was validated by qRT-PCR (**H**) and immunoblotting (**I**). (**J**) Stable EEF1D-overexpressing and vector control BCBL1 cells were treated with TPA for 48 h, and the transcript levels of representative KSHV lytic genes and the latent gene *ORF73* were measured using qRT-PCR. (**K**) Protein levels of RTA, EEF1D, and Tubulin in the same cells as in panel J were measured by immunoblotting. (**L and M**) Intracellular and extracellular KSHV DNA copy numbers were quantified using qPCR after treatment with TPA for 72 h. Data represent mean ± SD. Statistical significance was determined using an unpaired Student’s *t*-test. **, *P* < 0.01; ***, *P* < 0.001; ****, *P* < 0.0001.

Given that EEF1D interacts with RTA, the master regulator of KSHV lytic reactivation, we investigated whether EEF1D influences viral reactivation. To this end, iSLK.RGB cells were transduced with lentiviral particles expressing Flag-EEF1D or an empty vector, generating iSLK.RGB-Flag-EEF1D and iSLK.RGB-Flag-vector cell lines, respectively. Quantitative real-time PCR (qRT-PCR) and immunoblot analyses confirmed robust EEF1D overexpression in modified cells (Fig. 2A and B), thereby validating the successful establishment of the model system. Upon Dox-induced reactivation, qRT-PCR revealed that the transcription of representative viral lytic genes, as well as the latent gene *ORF73*, was significantly downregulated in EEF1D-overexpressing cells (Fig. 2C). Concurrently, the protein levels of RTA were markedly reduced in EEF1D-overexpressing cells compared to those in the control cells (Fig. 2D). Consistently, both intracellular and extracellular viral DNA copy numbers were significantly decreased (Fig. 2E and F). Furthermore, infection of HEK293T cells with progeny virions demonstrated that culture supernatants from EEF1D-overexpressing cells contained fewer infectious particles, as evidenced by the reduction in RFP-positive HEK293T cells (Fig. 2G).

To corroborate these findings, stable EEF1D-overexpressing and control cell lines were generated in the KSHV-positive BCBL1 background (Fig. 2H and I). Following TPA-induced reactivation, EEF1D overexpression similarly resulted in decreased expression of both the latent gene *ORF73* and lytic genes, attenuated RTA protein expression, lower total intracellular KSHV DNA copies, and reduced virion release compared to the vector control (Fig. 2J through M). Together, these results demonstrate that ectopic EEF1D expression suppresses KSHV lytic replication.

### Knockdown of endogenous EEF1D enhances KSHV lytic reactivation

**Fig 3:**
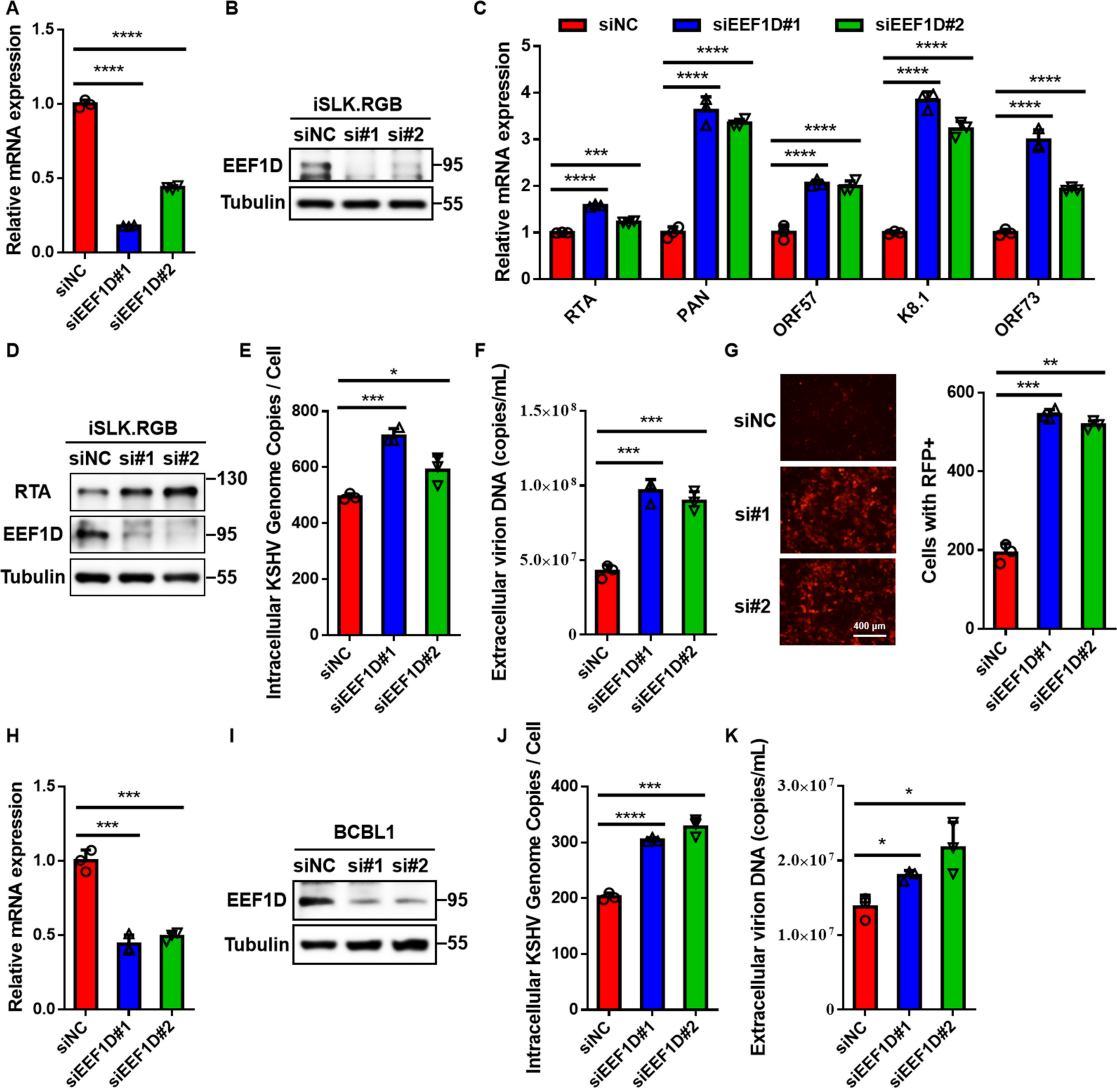
Knockdown of endogenous EEF1D enhances KSHV lytic replication. (**A and B**) iSLK.RGB cells were transfected with two distinct siRNAs targeting *EEF1D* or negative control siRNA (siNC) for 48 h. Knockdown efficiency was verified by qRT-PCR (**A**) and immunoblotting (**B**). (**C**) iSLK.RGB cells were transfected with *EEF1D*-specific siRNAs or siNC for 6 h, followed by Dox induction for 72 h. The transcript levels of representative KSHV lytic genes (*RTA, PAN, ORF57, and K8.1*) and the latent gene *ORF73* were quantified using qRT-PCR. (**D**) Immunoblot analysis of RTA, EEF1D, and Tubulin in cell lysates prepared as described in panel **C**. (**E and F**) Intracellular (**E**) and extracellular (**F**) KSHV DNA copy numbers in the same cells as in panel C were measured using qPCR. Intracellular viral load was expressed as copies per cell, and extracellular viral load was expressed as copies per mL. (**G**) Culture supernatants (1 mL) collected 96 h post-induction were used to infect HEK293T cells. At 24 h post-infection, RFP expression was visualized using fluorescence microscopy. Images were segmented using Cellpose, and the number of RFP-positive cells was quantified using the ImageJ software. (**H and I**) BCBL1 cells were transfected twice with two independent siRNAs targeting *EEF1D* or siNC at 24 h intervals to enhance knockdown efficiency. Knockdown efficiency was confirmed by qRT-PCR (**H**) and immunoblotting (**I**). (**J and K**) Following transfection, the cells were treated with TPA for 72 h. Intracellular and extracellular KSHV DNA copy numbers were quantified using qPCR. Viral loads are expressed as described in panels E and F. Data are presented as the mean ± SD. Statistical significance was determined using an unpaired Student’s *t*-test. *, *P* < 0.05; **, *P* < 0.01; ***, *P* < 0.001; ****, *P* < 0.0001.

To further define the role of EEF1D in the regulation of KSHV replication, we conducted loss-of-function analyses in iSLK.RGB cells. Cells were transfected with two independent small interfering RNAs (siRNAs) targeting *EEF1D* or a non-targeting control siRNA (siNC). qRT-PCR and immunoblot analyses confirmed the efficient knockdown of EEF1D at both the transcript and protein levels (Fig. 3A and B). Six hours after siRNA transfection, Dox was added to induce RTA expression and trigger lytic reactivation. EEF1D depletion markedly enhanced the transcription of representative lytic genes and the latent gene *ORF73* and increased RTA protein levels, accompanied by elevated intracellular viral genomic DNA loads and extracellular virion-associated DNA copy numbers (Fig. 3C through F). Furthermore, *de novo* infection assays demonstrated that culture supernatants from EEF1D-depleted cells resulted in a higher percentage of RFP-positive HEK293T cells, indicating increased production of infectious progeny virions (Fig. 3G).

Consistent results were obtained in BCBL1 cells, where lytic reactivation was induced by TPA treatment. Knockdown of EEF1D in this system also resulted in greater viral DNA amplification and enhanced virion release compared to the controls (Fig. 3H through K). Collectively, these findings establish that endogenous EEF1D acts as a negative regulator of KSHV lytic reactivation.

### KSHV RTA antagonizes the antiviral factor EEF1D by promoting its downregulation

**Fig 4:**
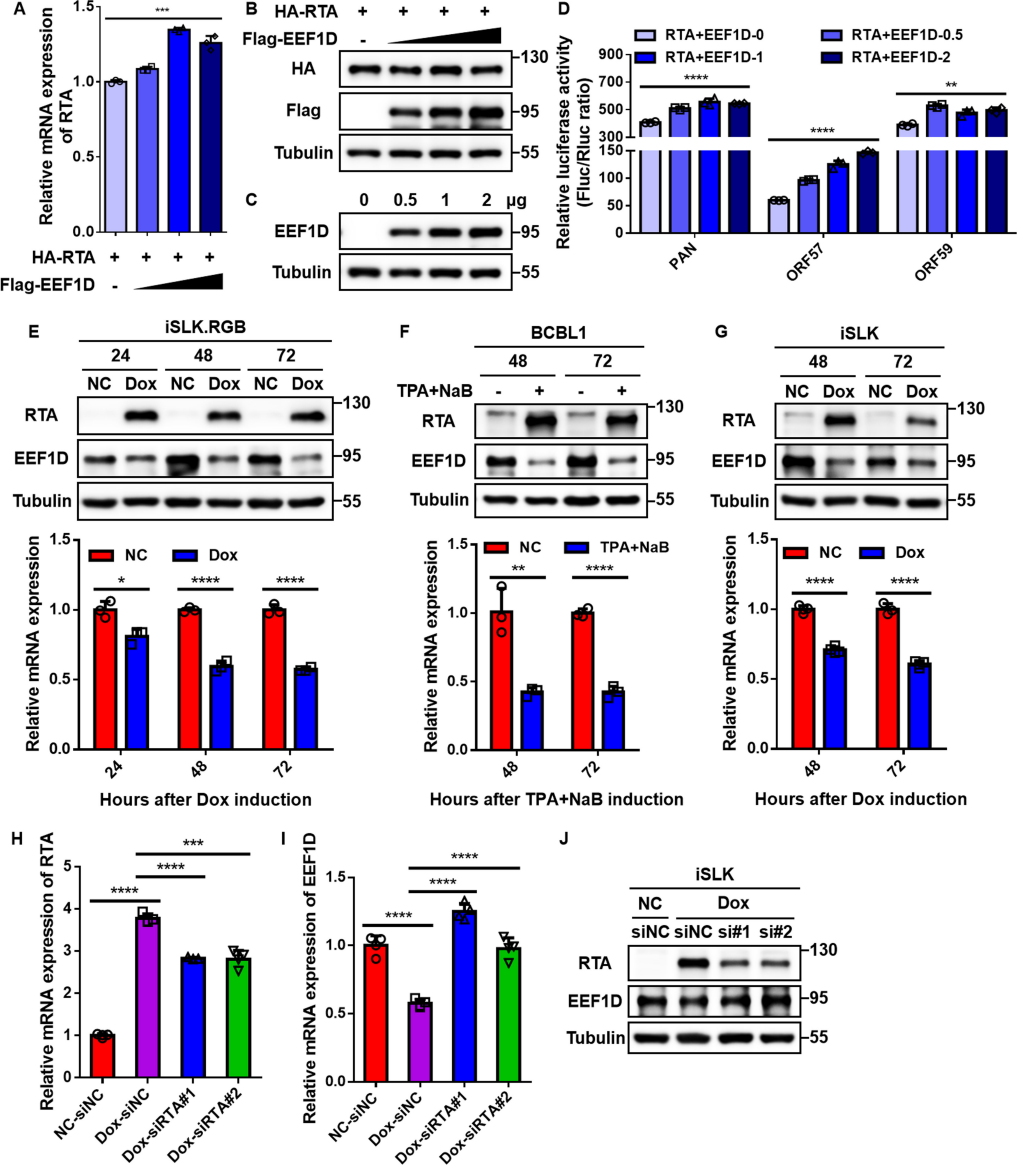
KSHV RTA antagonizes the antiviral factor EEF1D by promoting its downregulation. (**A and B**) Effect of EEF1D on RTA expression. HEK293T cells were co-transfected with HA-RTA and increasing amounts of Flag-EEF1D (0, 0.5, 1, and 2 μg) plasmids. At 48 h post-transfection, total RNA and whole-cell lysates were harvested. *RTA* mRNA levels were quantified using qRT-PCR (**A**), and protein levels were analyzed using immunoblotting (**B**). (**C**) Immunoblot confirmation of EEF1D expression levels in the luciferase reporter assays shown in panel **D**. (**D**) HEK293T cells were co-transfected with the indicated luciferase reporter plasmids (PANp, ORF57p, or ORF59p), pRL-TK (internal control), HA-RTA, and increasing amounts of Flag-EEF1D (0, 0.5, 1, and 2 μg). Luciferase activity was measured 48 h after transfection. (**E**) iSLK.RGB cells were treated with or without Dox (NC vs Dox) for the indicated times (24, 48, and 72 h). Protein levels of RTA, EEF1D, and Tubulin were analyzed by immunoblotting (upper panel). *EEF1D* mRNA levels were quantified by qRT-PCR and normalized to *GAPDH* levels (lower panel). (**F**) BCBL1 cells were treated with or without TPA (30 ng/mL) and sodium butyrate (NaB, 1 mM) for 48 h and 72 h. Protein levels were analyzed by immunoblotting (upper panel), and *EEF1D* mRNA levels were quantified using qRT-PCR (lower panel). (**G**) KSHV-negative iSLK cells (stably expressing Dox-inducible RTA) were treated with or without Dox for 48 h and 72 h. Protein levels of RTA, EEF1D, and Tubulin were analyzed by immunoblotting (upper panel). *EEF1D* mRNA levels were quantified using qRT-PCR (lower panel). (H to J) iSLK cells were treated with or without Dox to induce RTA expression. Six hours post-treatment, the medium was replaced with fresh medium, and the cells were transfected with siRNAs targeting *RTA* (siRTA#1 and siRTA#2) or negative control siRNA (siNC). At 48 h post-transfection, the cells were harvested. (**H**) The knockdown efficiency of *RTA* was confirmed using qRT-PCR. (**I**) *EEF1D* mRNA levels were quantified using qRT-PCR. (**J**) Protein levels of RTA, EEF1D, and Tubulin were analyzed by immunoblotting. Data represent mean ± SD. Statistical significance was determined using an unpaired Student’s *t*-test. *, *P* < 0.05; **, *P* < 0.01; ***, *P* < 0.001; ****, *P* < 0.0001.

Given that EEF1D interacts with RTA and suppresses KSHV lytic replication, we hypothesized that EEF1D inhibits viral reactivation by targeting RTA. To test this possibility, we examined whether EEF1D modulates RTA stability and transcriptional activity. Surprisingly, increasing EEF1D levels did not reduce RTA protein abundance (Fig. 4B), despite the modest accumulation of *RTA* mRNA (Fig. 4A). We further assessed the RTA transactivation function using luciferase reporter assays. The results showed that EEF1D modestly enhanced, rather than suppressed, RTA-mediated activation of the *PAN*, *ORF57*, and *ORF59* promoters (Fig. 4D). Immunoblot analysis confirmed the dose-dependent expression of EEF1D in these assays (Fig. 4C). Collectively, these data suggest that the suppressive effect of EEF1D on KSHV reactivation is not attributable to the direct inhibition of RTA expression or transcriptional activity.

Having excluded the possibility that EEF1D directly impairs RTA, we next focused on the interplay between KSHV and EEF1D during the lytic cycle. Given that EEF1D suppresses KSHV lytic replication, we hypothesized that KSHV downregulates EEF1D to overcome this suppression. To test this, iSLK.RGB cells were treated with Dox to induce lytic reactivation, and EEF1D levels were assessed at specific time points (24, 48, and 72 h). Immunoblot analysis revealed that EEF1D protein levels were markedly lower in Dox-induced cells than in their respective uninduced controls (NC), particularly at 48 and 72 h post-induction, a trend accompanied by the robust accumulation of RTA (Fig. 4E, upper panel). Consistent with the protein data, qRT-PCR analysis showed that *EEF1D* mRNA levels were significantly decreased in reactivated cells compared to time-matched controls (Fig. 4E, lower panel). To validate this phenomenon in naturally infected cells, we examined EEF1D expression in BCBL1 cells treated with TPA and sodium butyrate (NaB). Similarly, lytic reactivation led to a marked reduction in both EEF1D protein and mRNA levels at 48 and 72 h post-induction (Fig. 4F). Collectively, these findings indicate that KSHV lytic reactivation triggers the downregulation of EEF1D expression, suggesting a viral counter-defense mechanism to overcome EEF1D-mediated suppression.

Based on the physical interaction between EEF1D and RTA (Fig. 1), the established E3 ubiquitin ligase activity of RTA (33–37), and the observation that EEF1D is downregulated upon lytic reactivation (Fig. 4E and F), we hypothesized that RTA might drive this effect. To determine whether RTA alone is sufficient to modulate EEF1D expression in the absence of other viral proteins, we used KSHV-negative iSLK cells harboring a Dox-inducible RTA cassette (55). Upon Dox treatment, the induction of RTA expression triggered a robust decline in endogenous EEF1D protein levels (Fig. 4G, upper panel). Interestingly, *EEF1D* mRNA levels were also significantly downregulated following RTA induction (Fig. 4G, lower panel), suggesting that RTA exerts its suppressive effect through a dual mechanism targeting both EEF1D protein stability and transcript abundance. To further validate that this downregulation was strictly RTA-dependent, we performed rescue experiments using RNA interference. Dox-induced iSLK cells were transfected with siRNAs targeting *RTA* (siRTA) or control siRNA (siNC). The knockdown efficiency of *RTA* was confirmed using qRT-PCR (Fig. 4H). In the control group (siNC), Dox induction caused a significant reduction in EEF1D expression. However, RTA depletion effectively rescued both *EEF1D* mRNA levels (Fig. 4I) and protein abundance (Fig. 4J). Collectively, these findings demonstrate that RTA is necessary and sufficient to downregulate EEF1D expression.

### RTA promotes EEF1D ubiquitination and degradation, yet mRNA downregulation dominates its reduction

Given the direct physical interaction between RTA and EEF1D (Fig. 1) and the well-documented E3 ubiquitin ligase activity of RTA (33–37), we hypothesized that RTA regulates EEF1D protein stability. Concurrently, RTA also reduced *EEF1D* mRNA levels (Fig. 4G through J). These observations suggest that RTA employs a dual regulatory mechanism that targets both protein stability and transcript abundance.

**Fig 5:**
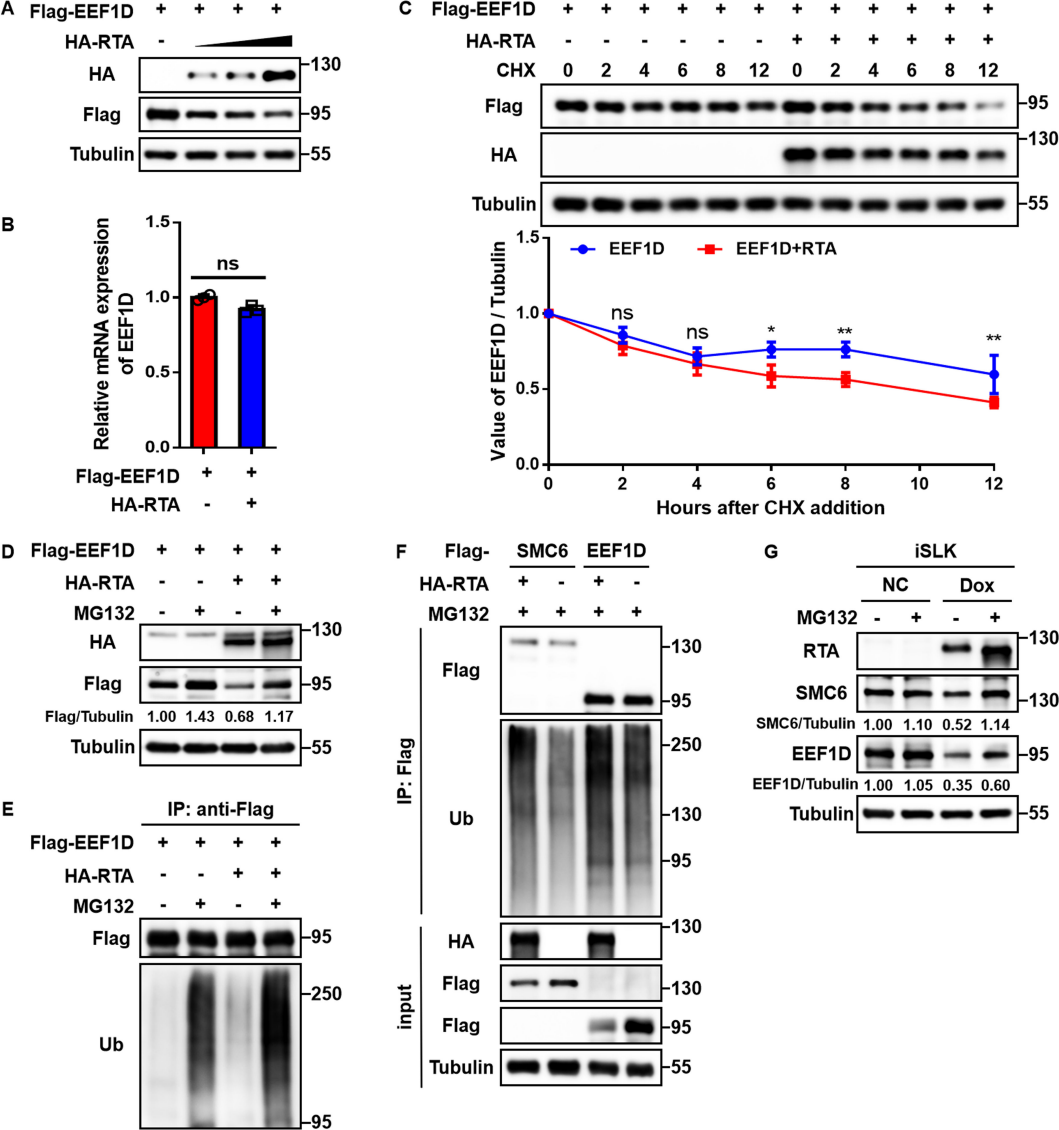
RTA promotes EEF1D ubiquitination and degradation, but mRNA downregulation dominates its reduction. (**A**) HEK293T cells were transfected with Flag-EEF1D and increasing amounts of HA-RTA (0, 0.5, 1, and 2 μg) for 48 h. Cell lysates were subjected to immunoblotting using the indicated antibodies. (**B**) RT-qPCR analysis of *EEF1D* mRNA levels in HEK293T cells transfected with Flag-EEF1D ± HA RTA. (**C**) HEK293T cells expressing Flag-EEF1D ± HA RTA were treated with cycloheximide (CHX, 100 μg/mL) 36 h post-transfection for the indicated times. Protein levels were analyzed by immunoblotting (top panel), and relative EEF1D abundance is shown in the bottom panel. (**D**) HEK293T cells expressing Flag-EEF1D and HA-RTA were treated with DMSO or MG132 (10 μM) for 10 h before harvesting. The lysates were analyzed using immunoblotting. Relative protein intensities are indicated below each band. (**E**) Lysates from cells treated as in (**D**) were immunoprecipitated with anti-Flag M2 affinity gel and analyzed by immunoblotting using the indicated antibodies. (**F**) Ubiquitination assay comparing SMC6 and EEF1D. HEK293T cells were transfected with the indicated plasmids (Flag-SMC6 or Flag-EEF1D, with or without HA-RTA). Thirty-six hours post-transfection, the cells were treated with MG132 (10 μM) for another 10 h. Lysates were immunoprecipitated with anti-Flag M2 affinity gel and immunoblotted with antibodies against the indicated proteins. (**G**) iSLK cells were treated with Dox to induce RTA expression or were left untreated (NC). Thirty-six hours later, the cells were treated with DMSO or MG132 (10 μM) for 10 h. Endogenous protein levels were analyzed using immunoblotting. Relative protein intensities are shown below each blot. For all quantitative immunoblots, band intensities were quantified using ImageQuant TL software and normalized to Tubulin. Data are presented as the mean ± SD. Statistical significance was assessed using an unpaired Student’s *t*-test. ns, not significant; *, *P* < 0.05; **, *P* < 0.01.

To dissect these mechanisms, we first assessed the effect of RTA on EEF1D protein stability. In an overexpression system designed to isolate protein stability from endogenous transcript regulation, ectopic RTA expression induced a dose-dependent reduction in EEF1D protein levels (Fig. 5A). Notably, RT-qPCR analysis confirmed that the transcript levels of *EEF1D* remained constant (Fig. 5B), indicating that the observed reduction in this system occurred strictly at the post-translational level.

To determine whether this post-translational regulation is specifically mediated by the ubiquitin-proteasome pathway, we performed a series of mechanistic assays. Cycloheximide (CHX) chase assays revealed that RTA expression significantly shortened the half-life of EEF1D (Fig. 5C). Consistently, this downregulation was effectively blocked by the proteasome inhibitor MG132 (Fig. 5D). Furthermore, ubiquitination assays demonstrated that RTA robustly promoted the polyubiquitination of EEF1D (Fig. 5E). To validate this specific regulation, we performed a side-by-side comparison with the chromatin maintenance factor SMC6, a known RTA substrate that is degraded via the proteasome without transcriptional changes (37). The results showed that RTA induced a similar polyubiquitination pattern on EEF1D as it did on SMC6 (Fig. 5F). Collectively, these findings confirm the involvement of the ubiquitin-proteasome pathway and establish that RTA targets EEF1D for degradation.

With this degradation mechanism established, its relative contribution to the total downregulation of endogenous EEF1D was evaluated in the iSLK cells. Given that RTA induction significantly reduced *EEF1D* mRNA levels (Fig. 4), we sought to dissect the specific impact of post-translational instability. Dox-induced RTA expression caused a robust decline in endogenous EEF1D protein levels (Fig. 5G, lane 1 vs lane 3). To assess the extent to which proteasomal degradation drives this phenotype, the cells were treated with MG132. SMC6, the established reference for purely proteasome-dependent regulation, was completely restored by MG132 (Fig. 5G, middle panel), confirming effective proteasome inhibition. In contrast, EEF1D protein levels were only partially restored by MG132 (increased from 0.35 to 0.60 relative to control) (Fig. 5G, bottom panel). The inability of proteasome inhibition to fully rescue EEF1D indicates that while the ubiquitin-proteasome pathway contributes to regulation, the reduction in transcript abundance remains the predominant mechanism underlying EEF1D downregulation following RTA induction.

Taken together, these data delineate a dual mechanism of interference: while RTA directly destabilizes EEF1D protein via the ubiquitin-proteasome pathway, the significant downregulation of *EEF1D* transcripts acts as a critical limiting factor that prevents the restoration of EEF1D protein levels after RTA induction.

### RTA-mediated repression of *EEF1D* promoter is conserved among primate γ-herpesviruses

**Fig 6:**
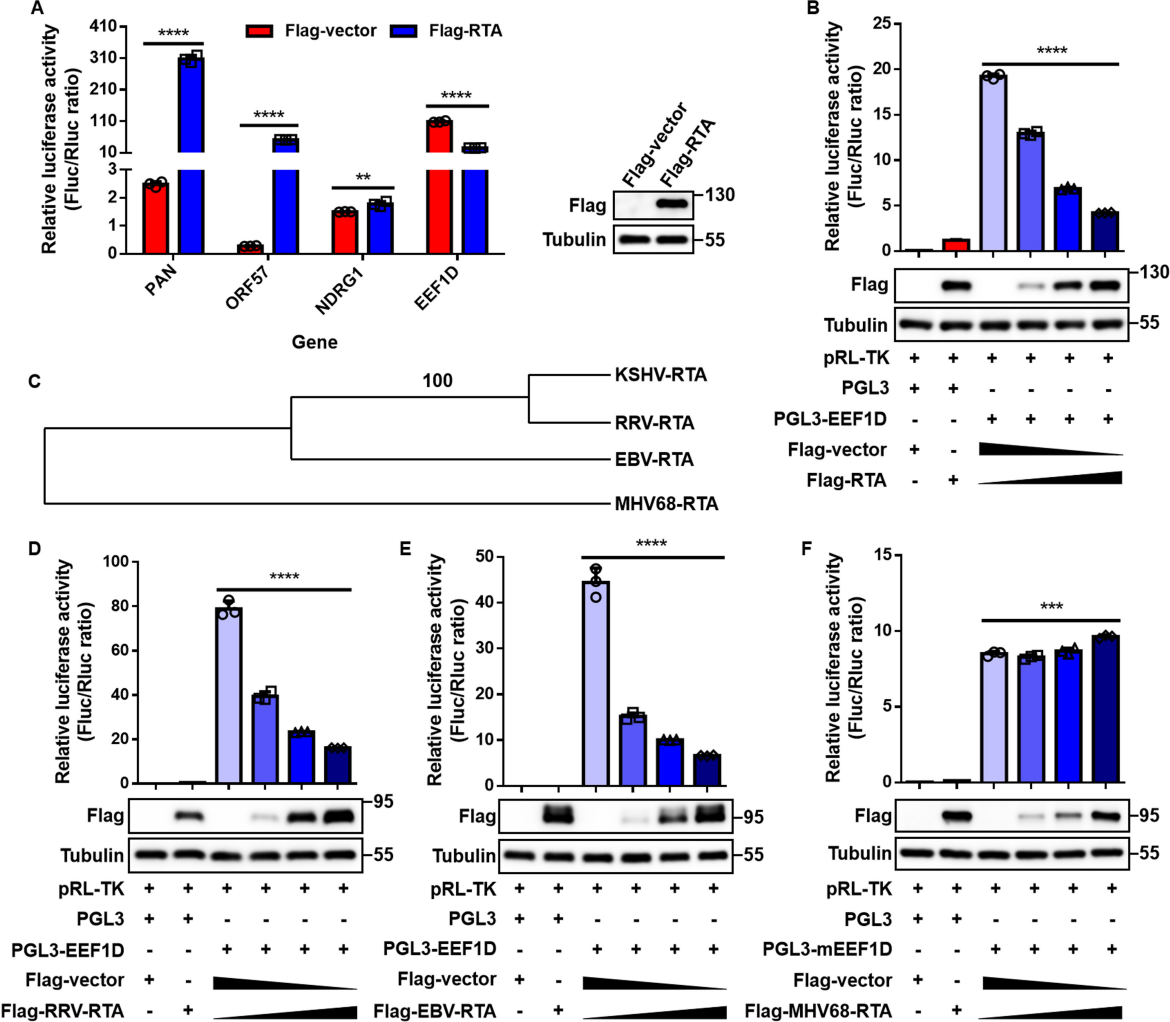
RTA functions as a transcriptional repressor of *EEF1D*, conserved in KSHV, RRV, and EBV, but not in MHV68. (**A**) Dual-luciferase reporter assays examining the effects of RTA on the promoters of *PAN*, *ORF57*, *NDRG1*, and *EEF1D* genes. HEK293T cells were co-transfected with promoter-firefly luciferase constructs (*PAN*, *ORF57*, *NDRG1*, or *EEF1D*), pRL-TK Renilla luciferase plasmid, and either Flag-RTA or an empty vector control. After 48 h of transfection, luciferase activity and protein expression were analyzed. (**B**) HEK293T cells were co-transfected with the *EEF1D* promoter construct and increasing amounts of RTA expression plasmid. Luciferase activity and protein levels were assessed 48 h after transfection. (**C**) Phylogenetic tree of RTA homologs from KSHV, RRV, EBV, and MHV68 constructed using MEGA. (**D–F**) HEK293T cells were co-transfected with the *EEF1D* promoter construct and Flag-RTA homologs from RRV, EBV, and MHV68. At 48 h post-transfection, luciferase activity and protein expression were measured. In panel F, “pGL3-mEEF1D” contains the mouse *EEF1D* promoter (“m” denotes mouse). Data are shown as the mean ± SD. Statistical significance was assessed by unpaired Student’s *t*-test. **, *P* < 0.01; ***, *P* < 0.001; ****, *P* < 0.0001.

The above findings demonstrated that RTA reduces EEF1D expression, mainly at the transcript level. Given that RTA is best known as a potent transcriptional activator (25–32), and that some transcription factors exhibit context-dependent dual roles as activators or repressors depending on cellular context and cofactors (43–46), we further explored the possibility that RTA functions as a transcriptional repressor of *EEF1D*. To test this, the *EEF1D* promoter region (−2,095 to +395 relative to the transcription start site) was cloned into a luciferase reporter construct. Dual-luciferase assays revealed that RTA markedly repressed *EEF1D* promoter activity (Fig. 6A). In contrast, known RTA-responsive promoters, such as *PAN* and *ORF57* (KSHV lytic genes), and *NDRG1* (a cellular control) (56), were activated under the same conditions (Fig. 6A), indicating that the repression of *EEF1D* was specific. A dose-response analysis further revealed a progressive reduction in *EEF1D* promoter activity with increasing RTA expression (Fig. 6B).

Because RTA is conserved across γ-herpesviruses and retains core transcriptional functions (57), we investigated whether this repressive activity toward *EEF1D* is evolutionarily conserved. Phylogenetic comparison of RTA homologs from KSHV, EBV, RRV, and MHV68 indicated that RRV-RTA and EBV-RTA are closely related to KSHV-RTA, whereas MHV68-RTA is more divergent (Fig. 6C). To test for functional conservation, we co-transfected an *EEF1D* promoter-luciferase reporter with RTA homologs from each virus. RTA proteins from KSHV, RRV, and EBV suppressed *EEF1D* promoter activity in a dose-dependent manner, whereas MHV68-RTA enhanced its activity (Fig. 6D through F).

Together, these findings establish that RTA acts as a transcriptional repressor of *EEF1D*, and that this function is conserved among primate γ-herpesviruses but absent in murine MHV68.

### RTA-mediated repression requires DNMT-dependent promoter hypermethylation

**Fig 7:**
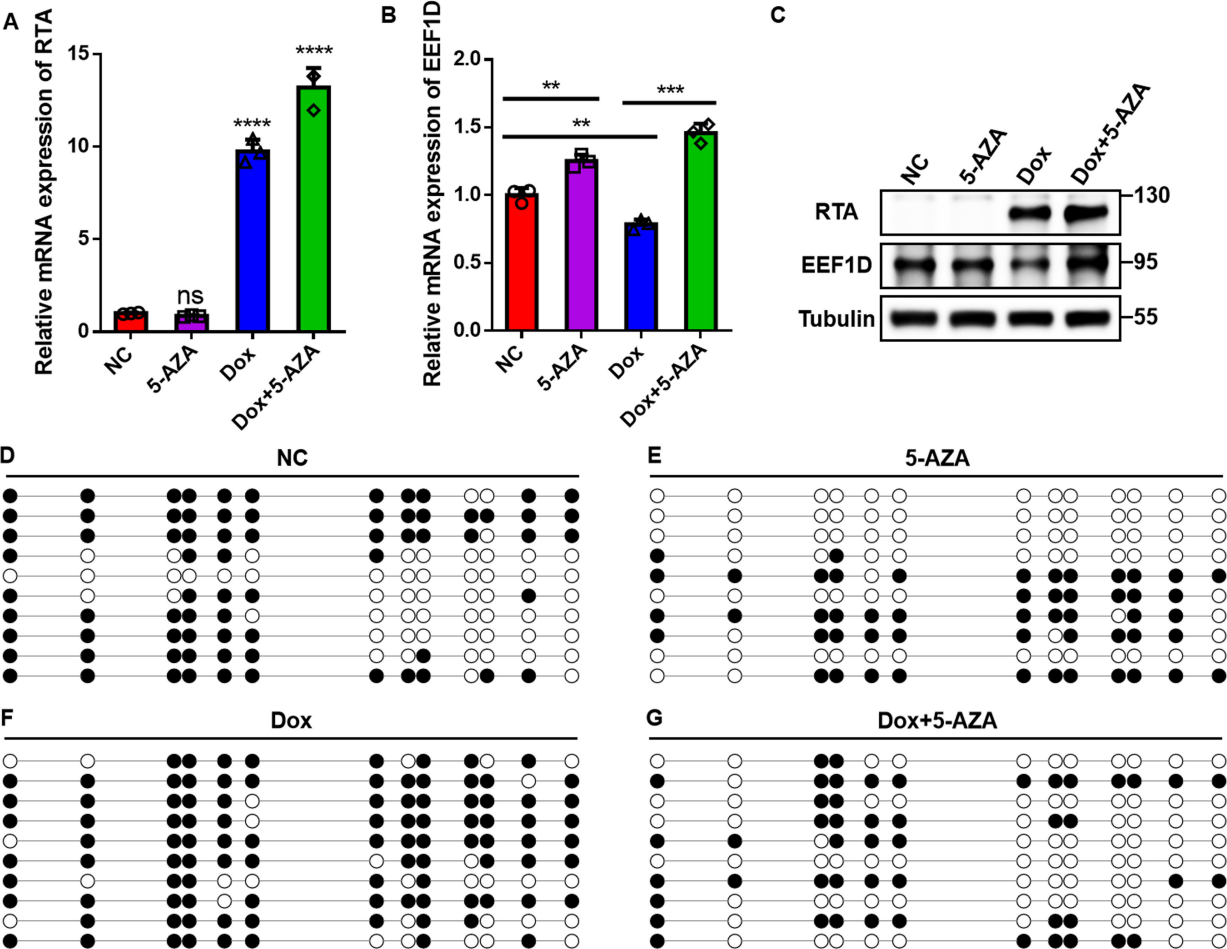
RTA-mediated repression requires DNMT-dependent promoter hypermethylation. (**A–C**) iSLK cells were treated with Dox, 5-AZA (5 μM), both, or left untreated (negative control, NC). EEF1D and RTA expression was assessed using qRT-PCR and immunoblotting after 48 h. (**D–G**) Bisulfite sequencing PCR (BSP) analysis of the *EEF1D* promoter in cells treated as described in panels **A–C**. Each row represents a DNA clone; open and filled circles indicate unmethylated and methylated CpG sites, respectively. Data are shown as the mean ± SD. Statistical significance was assessed by unpaired Student’s *t*-test. ns, not significant; **, *P* < 0.01; ***, *P* < 0.001; ****, *P* < 0.0001.

Our findings showed that RTA represses *EEF1D* transcription; however, the underlying mechanism remains unclear. Given that promoter DNA methylation is a well-known mechanism of transcriptional silencing, we examined whether RTA suppresses EEF1D expression through DNA methyltransferase (DNMT)-dependent promoter hypermethylation. To test this, iSLK cells were treated with Dox in the presence or absence of the DNMT inhibitor 5-aza-2’-deoxycytidine (5-AZA). After 48 h, qRT-PCR and immunoblotting showed that Dox-induced RTA expression reduced EEF1D transcript and protein levels, whereas co-treatment with 5-AZA restored the expression to near baseline levels (Fig. 7A through C), indicating that the repression is DNMT-dependent.

To directly assess DNA methylation at the *EEF1D* promoter, we performed bisulfite sequencing PCR (BSP). Cells treated with Dox alone displayed a marked increase in promoter methylation compared to untreated controls (Fig. 7D and F), indicating that RTA promotes *EEF1D* promoter hypermethylation. In contrast, cells co-treated with Dox and 5-AZA showed a substantial reduction in promoter methylation, comparable to that observed with 5-AZA treatment alone (Fig. 7E and G), confirming that DNMT activity is required for RTA-induced promoter methylation.

Collectively, these findings demonstrate that KSHV RTA represses *EEF1D* transcription by inducing promoter hypermethylation in a manner dependent on host DNA methyltransferase activity.

### DNMT3A specifically mediates RTA-induced promoter hypermethylation

**Fig 8:**
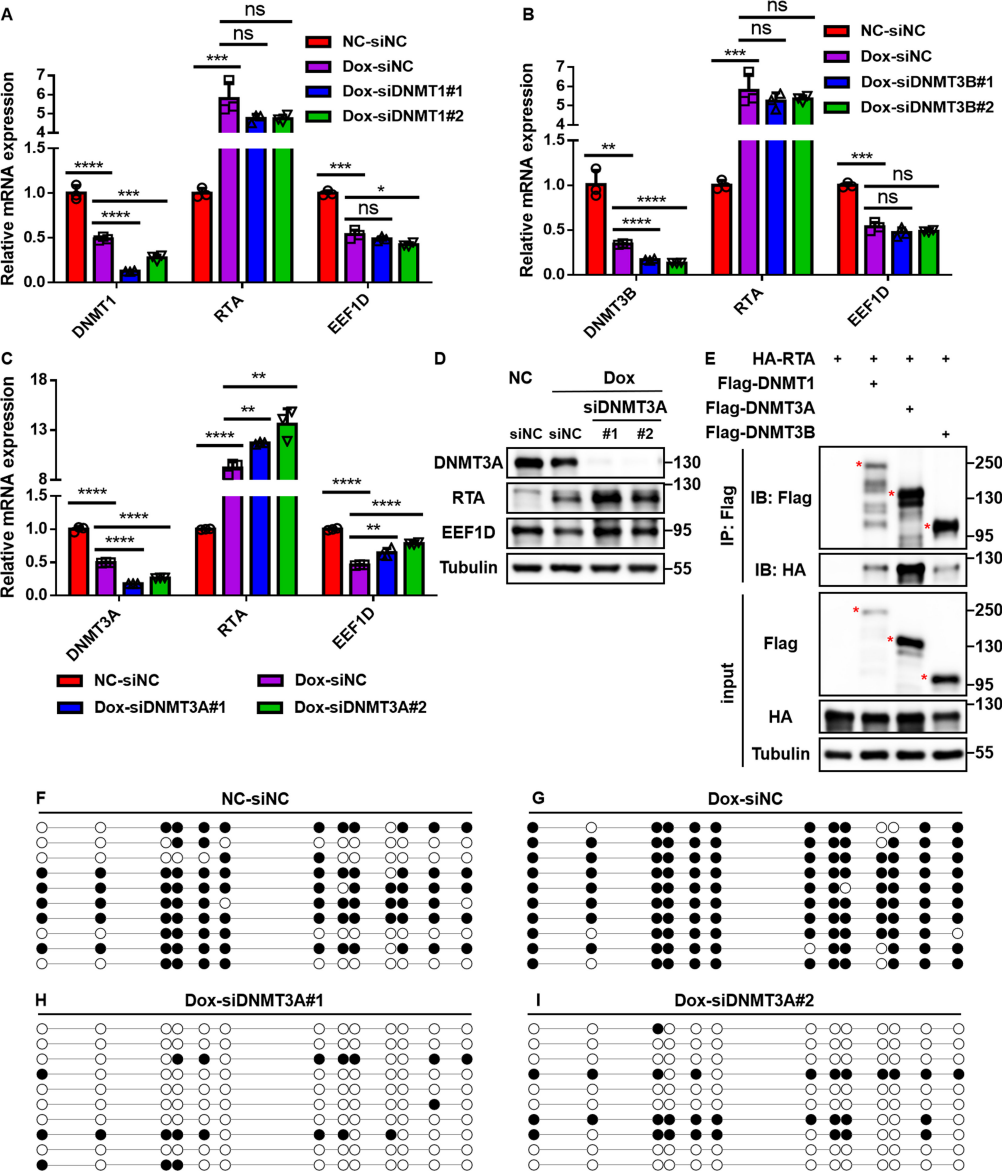
DNMT3A mediates RTA-induced hypermethylation of *EEF1D* promoter. (**A–C**) iSLK cells were left untreated or treated with Dox to induce RTA expression, followed by transfection with two distinct siRNAs targeting DNMT1, DNMT3A, or DNMT3B, or a control siRNA for 48 h. The transcript levels of DNMTs, RTA, and EEF1D were analyzed using qRT-PCR. (**D**) iSLK cells were treated as described in (**C**), and the protein levels of DNMT3A, RTA, and EEF1D were examined by immunoblotting. (**E**) HEK293T cells were co-transfected with HA-RTA and Flag-DNMT1, DNMT3A, or DNMT3B. Cell lysates were immunoprecipitated with anti-Flag M2 beads and analyzed by immunoblotting. (**F–I**) BSP analysis of the *EEF1D* promoter in iSLK cells treated as described in panel **C**. Each row represents one DNA clone. Open and filled circles denote unmethylated and methylated CpG sites, respectively. Data are presented as mean ± SD. Statistical significance was determined by unpaired Student’s *t*-test. ns, not significant; *, *P* < 0.05; **, *P* < 0.01; ***, *P* < 0.001; ****, *P* < 0.0001.

The above results demonstrate that KSHV RTA represses *EEF1D* transcription through DNMT-dependent promoter hypermethylation. Given that promoter hypermethylation in mammalian cells is primarily mediated by DNMT1, DNMT3A, and DNMT3B (58), we selectively silenced each of these enzymes in Dox-induced iSLK cells. In cells transfected with control siRNA, RTA was robustly induced, and *EEF1D* expression was markedly downregulated, confirming effective transcriptional repression (Fig. 8A through C). Notably, knockdown of DNMT3A, but not DNMT1 or DNMT3B, restored *EEF1D* transcript levels despite sustained RTA expression, and this recovery was further validated at the protein level (Fig. 8A through D), indicating that DNMT3A specifically mediates the RTA-induced repression of *EEF1D*. To determine whether DNMT3A physically associates with RTA, Co-IP assays were performed. RTA preferentially interacted with DNMT3A compared to the other *de novo* methyltransferase DNMT3B (Fig. 8E), suggesting the selective recruitment of DNMT3A during lytic reactivation. BSP analysis further confirmed this mechanism: Dox-induced RTA expression caused marked hypermethylation of the *EEF1D* promoter relative to untreated controls (Fig. 8F and G), whereas silencing DNMT3A substantially reduced RTA-induced hypermethylation (Fig. 8G through I). Collectively, these results identify DNMT3A as the key methyltransferase recruited by RTA to repress EEF1D transcription via promoter hypermethylation.

### PATZ1 mediates RTA-induced epigenetic silencing of *EEF1D*

**Fig 9:**
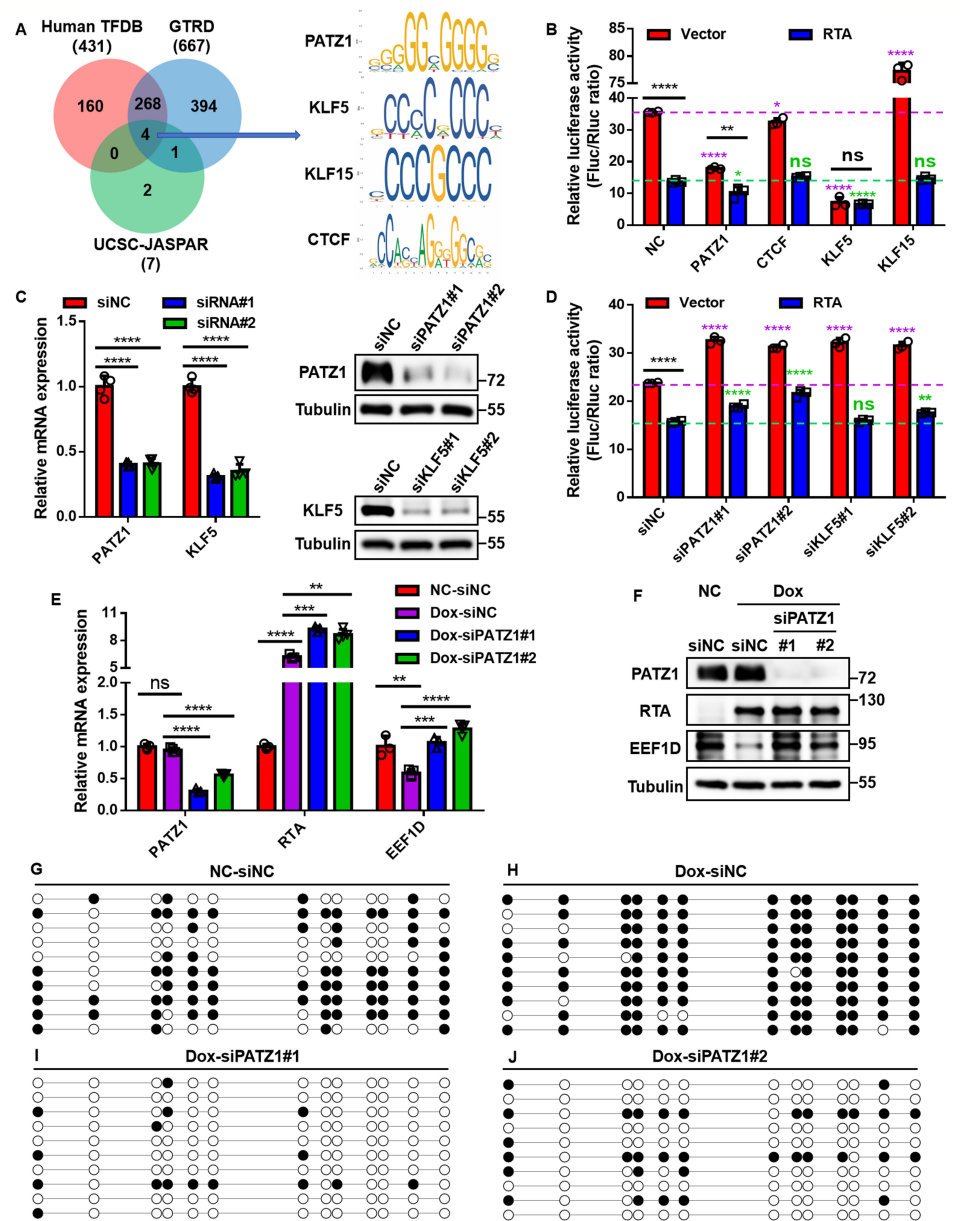
Identification of PATZ1 as a mediator of RTA-induced regulation of the *EEF1D* promoter. (**A**) Candidate transcription factors predicted to bind the *EEF1D* promoter using human TFDB, GTRD, and UCSC-JASPAR databases; overlaps were visualized using a Venn diagram. (**B**) Dual-luciferase reporter assays in HEK293T cells co-transfected with the *EEF1D* promoter construct, pRL-TK, HA-RTA or vector control, and candidate transcription factors (PATZ1, CTCF, KLF5, and KLF15). Luciferase activity was measured 48 h after transfection. (**C**) HEK293T cells were transfected with two distinct siRNAs targeting PATZ1 or KLF5 or with a control siRNA. Knockdown efficiency was validated using qRT-PCR and immunoblotting. (**D**) Effect of PATZ1 or KLF5 knockdown on RTA-mediated repression of the *EEF1D* promoter activity. HEK293T cells were first transfected with the indicated siRNAs, followed by transfection with the EEF1D promoter reporter construct, pRL-TK, and HA-RTA or vector control 6 h later. Luciferase activity was then measured. (**E and F**) Analysis of endogenous EEF1D expression in iSLK cells (inducible RTA). Cells were transfected with siRNAs targeting PATZ1 (siPATZ1#1 or siPATZ1#2) or a negative control (siNC), followed by treatment with Dox to induce RTA expression. *EEF1D* and *RTA* levels were determined by RT-qPCR (**E**) and immunoblotting (**F**). (**G–J**) BSP analysis of *EEF1D* promoter methylation status. Cells were treated as described in panels **E and F**. Each row represents one DNA clone. Filled circles represent methylated CpG sites; open circles represent unmethylated CpG sites. Data are presented as mean ± SD. Statistical significance was determined by unpaired Student’s *t*-test. ns, not significant; *, *P* < 0.05; **, *P* < 0.01; ****, *P* < 0.0001.

The above results demonstrate that RTA represses *EEF1D* transcription by recruiting DNMT3A to induce promoter hypermethylation. However, because the *EEF1D* promoter lacks a canonical RTA-binding motif, we hypothesized that RTA might be recruited through cellular cofactors. To identify potential mediators, an *in silico* prediction was performed by intersecting the Human TFDB (59), GTRD (60), and UCSC-JASPAR (61) databases (Table S2). This comparative analysis identified four high-confidence candidate transcription factors, PATZ1, KLF5, KLF15, and CTCF, that possess binding motifs within the *EEF1D* promoter region (Fig. 9A).

To screen these candidates, dual-luciferase reporter assays were performed. Overexpression of PATZ1 or KLF5 significantly suppressed *EEF1D* promoter activities. Notably, co-expression of PATZ1 and RTA resulted in a further reduction in promoter activity compared to either factor alone (Fig. 9B). To identify the functional mediator, specific siRNAs targeting PATZ1 and KLF5 were employed (Fig. 9C). The results showed that depletion of PATZ1, but not KLF5, significantly attenuated RTA-induced repression of the *EEF1D* promoter (Fig. 9D), indicating that PATZ1 is specifically required for transcriptional suppression.

The role of PATZ1 was further validated in iSLK cells. While the induction of RTA expression by Dox led to a significant reduction in both *EEF1D* mRNA and protein levels, this downregulation was effectively reversed by PATZ1 knockdown (Fig. 9E and F). Finally, given that RTA induction leads to promoter hypermethylation, we investigated the involvement of PATZ1 in this epigenetic process. BSP analysis demonstrated that while RTA expression caused extensive CpG methylation at the *EEF1D* promoter, PATZ1 silencing abolished this effect. Surprisingly, PATZ1 depletion reduced the methylation frequency to a level lower than that of the latent control (Fig. 9G through J), suggesting that PATZ1 may contribute to the maintenance of basal promoter methylation, in addition to mediating RTA-induced silencing.

Collectively, these findings demonstrate that PATZ1 is indispensable for RTA-mediated transcriptional repression and is a critical regulator of *EEF1D* promoter methylation.

### PATZ1 recruits the RTA-DNMT3A complex to specific regulatory nodes on the *EEF1D* promoter

**Fig 10:**
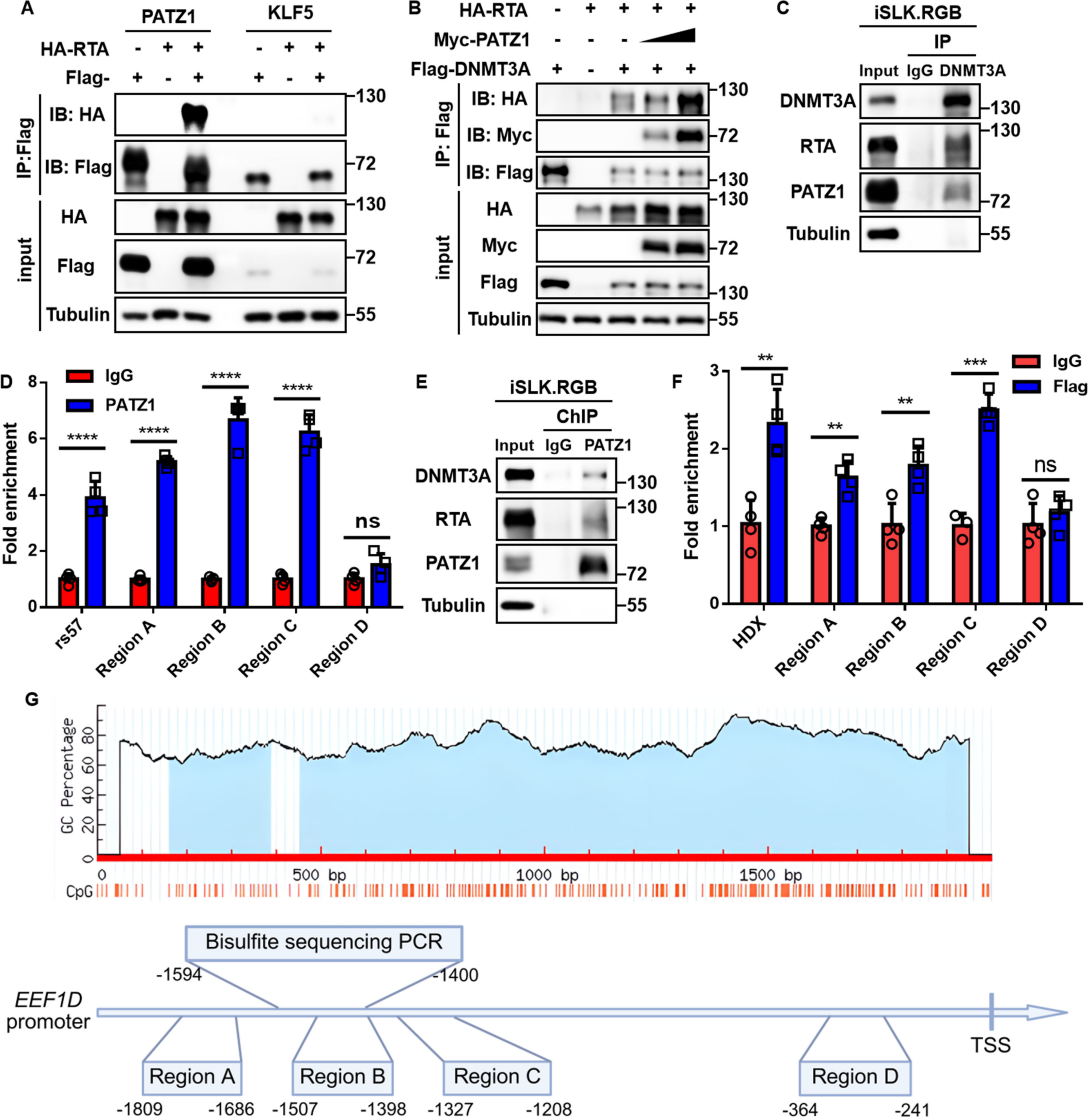
PATZ1 recruits the RTA-DNMT3A complex to specific regulatory nodes on the *EEF1D* promoter. (**A**) Co-IP analysis of the interaction between RTA and PATZ1. HEK293T cells were singly or co-transfected with Flag-PATZ1 (or the control Flag-KLF5) and HA-RTA expression plasmids. Cell lysates were collected 48 h post-transfection for Co-IP and immunoblotting. (**B**) Co-IP analysis of ternary complex formation in HEK293T cells co-transfected with Flag-DNMT3A, HA-RTA, and increasing amounts of Myc-PATZ1. (**C**) Endogenous Co-IP assay in iSLK.RGB cells. Cells were treated with Dox to induce lytic reactivation for 48 h. Lysates were immunoprecipitated with anti-DNMT3A antibody or IgG control, and interactions with RTA and PATZ1 were detected by immunoblotting. (**D**) ChIP-qPCR analysis of PATZ1 occupancy on the *EEF1D* promoter during viral reactivation. Chromatin from Dox-treated iSLK.RGB cells was immunoprecipitated with anti-PATZ1 antibody or IgG control. Enriched DNA was analyzed by qPCR using primers targeting the *EEF1D* promoter regions (**A–D**). The *rs57* locus served as a positive control, and region D was utilized as a positional control. (**E**) ChIP-immunoblot analysis. Chromatin from Dox-treated iSLK.RGB cells was immunoprecipitated with anti-PATZ1 antibody or IgG control. The presence of DNMT3A and RTA in the immunoprecipitates was analyzed using immunoblotting. (**F**) ChIP-qPCR analysis of RTA binding to *EEF1D* promoter. iSLK.RGB cells were transfected with Flag-RTA using the Fugene HD transfection reagent. At 48 h post-transfection, chromatin was immunoprecipitated with anti-Flag antibody or IgG control, followed by qPCR. The *HDX* promoter was used as a positive control for RTA binding, and region D served as a positional control. (**G**) Schematic representation of the *EEF1D* promoter. The GC content and CpG dinucleotide distribution (orange vertical bars) are shown in the upper panel. The lower diagram illustrates the relative genomic positions of the regions analyzed by ChIP-qPCR (regions A–D) and the region targeted for BSP. Numerical values indicate the nucleotide positions relative to the transcription start site (TSS). Data are presented as mean ± SD. Statistical significance was determined by unpaired Student’s *t*-test. ns, not significant; **, *P* < 0.01; ***, *P* < 0.001; ****, *P* < 0.0001.

PATZ1 (POZ/BTB and AT-hook-containing zinc finger protein 1) is an architectural transcription factor of the POZ domain Krüppel-like zinc finger (POK) family that typically functions as a molecular scaffold to recruit co-repressor complexes (62). Given these properties, we hypothesized that PATZ1 facilitates recruitment of the RTA-DNMT3A complex to the *EEF1D* promoter. To test this hypothesis, we first examined the physical interplay between RTA and PATZ1. Because KLF5 was also identified as a repressor of the *EEF1D* promoter but was dispensable for RTA-mediated silencing (Fig. 9), it was included as a specificity control. Co-IP assays in HEK293T cells revealed that RTA strongly interacted with PATZ1, whereas no interaction was observed with KLF5 (Fig. 10A), confirming the specificity of the RTA-PATZ1 interaction. Furthermore, Co-IP assays demonstrated that RTA, PATZ1, and DNMT3A form a ternary complex (Fig. 10B). This interaction was validated at the endogenous level in iSLK.RGB cells upon KSHV lytic cycle induction. Under these reactivated conditions, immunoprecipitation of DNMT3A successfully pulled down both RTA and PATZ1 (Fig. 10C). These results indicate that PATZ1 physically interacts with RTA and DNMT3A to form a stable ternary complex during KSHV lytic reactivation.

To determine whether the multi-protein complex assembles specifically on the *EEF1D* promoter, we performed chromatin immunoprecipitation (ChIP) assays during KSHV reactivation. Based on JASPAR predictions, we initially screened multiple primer sets covering the predicted PATZ1-binding clusters. Following systematic optimization, four representative regions (regions A–D) were selected for further analysis based on their high amplification specificity and consistent performance, characterized by single, sharp melting curve peaks. The precise genomic locations of these regions relative to the promoter architecture are illustrated in Fig. 10G. ChIP-qPCR analysis revealed robust PATZ1 occupancy at regions A, B, and C, as well as the positive control *rs57* (63). Notably, region D was utilized as a positional control; although it harbors predicted PATZ1-binding motifs and sits closer to the TSS (Fig. 10G), it exhibited no significant enrichment (Fig. 10D). After establishing PATZ1 binding, we investigated whether it recruits the repressor complex to the chromatin. Crucially, a ChIP-immunoblot analysis revealed that pulling down chromatin-bound PATZ1 resulted in the co-precipitation of both RTA and DNMT3A (Fig. 10E). These results confirmed that PATZ1 physically recruits the RTA-DNMT3A repressor complex to the *EEF1D* promoter region.

To substantiate this recruitment with direct RTA-binding data, we initially attempted to map endogenous RTA. However, owing to the lack of a ChIP-grade antibody and the infeasibility of generating stable cell lines due to RTA-induced lytic cell death, we adopted a transient transfection strategy. To this end, iSLK.RGB cells were transfected with Flag-RTA. Subsequent ChIP-qPCR analysis using an anti-Flag antibody demonstrated that Flag-RTA showed a similar occupancy pattern to that of PATZ1, with significant enrichment observed at regions A–C but not at region D (Fig. 10F), while the *HDX* promoter (64) served as a positive control. Remarkably, the binding profiles of PATZ1 and RTA correlate with the spatial arrangement of the *EEF1D* promoter (Fig. 10G). Regions A, B, and C, which exhibited robust enrichment, are located in close proximity to the BSP targeted region. In contrast, although region D resides within the same CpG island as regions B and C, it is positioned significantly closer to the TSS and further away from the BSP-targeted cluster (Fig. 10G). These spatial observations suggest that the assembly of the RTA-PATZ1-DNMT3A complex is localized to specific regulatory nodes flanking the methylation-sensitive region. Collectively, these data indicate that PATZ1 facilitates the site-specific recruitment of the RTA-DNMT3A complex to the *EEF1D* promoter.

### PATZ1 is indispensable for RTA-mediated *EEF1D* silencing and promoter hypermethylation during KSHV reactivation

**Fig 11:**
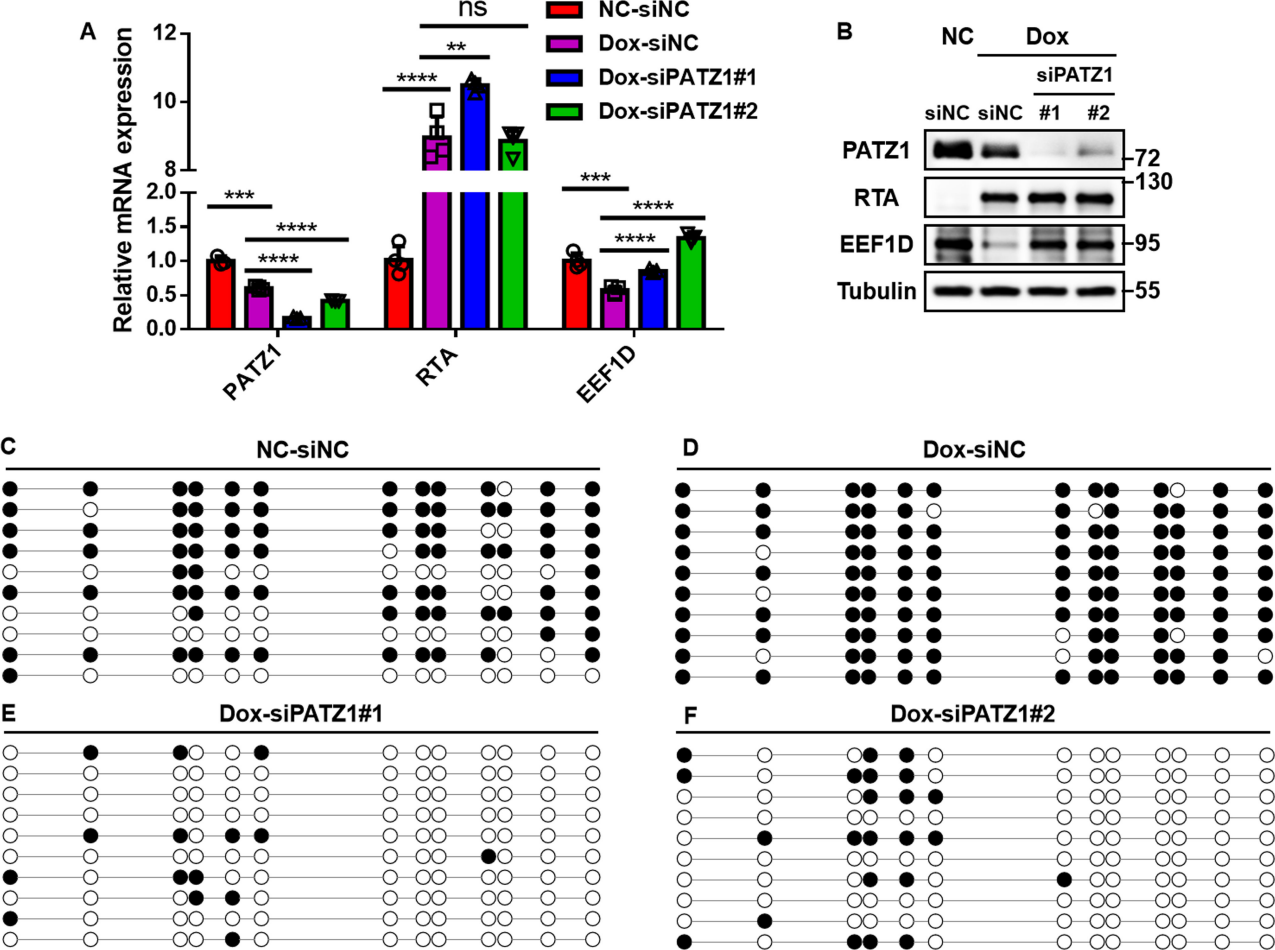
(**A and B**) Analysis of endogenous EEF1D expression in iSLK.RGB cells. Cells were transfected with siRNAs targeting PATZ1 or negative control (siNC), followed by Dox induction for 72 h. *EEF1D* and *RTA* levels were determined by RT-qPCR (**A**) and immunoblotting (**B**), respectively. (**C–F**) BSP analysis of *EEF1D* promoter methylation. Genomic DNA was extracted from iSLK.RGB cells representing latent infection (**C**), lytic reactivation (**D**), and lytic reactivation with PATZ1 knockdown (**E and F**). Filled and open circles represent methylated and unmethylated CpG sites, respectively. Data are presented as mean ± SD. Statistical significance was determined by unpaired Student’s *t*-test. ns, not significant; **, *P* < 0.01; ***, *P* < 0.001; ****, *P* < 0.0001.

Our preceding results established that PATZ1 mediates RTA-induced epigenetic silencing of *EEF1D* in KSHV-negative iSLK cells (Fig. 9) and physically bridges the RTA-DNMT3A complex to specific regulatory nodes of the *EEF1D* promoter (Fig. 10). To determine whether this mechanism holds true in the context of a complete viral life cycle, we performed a functional rescue and epigenetic analysis in iSLK.RGB cells undergoing KSHV lytic reactivation. Using the validated siRNAs that effectively depleted PATZ1 (Fig. 9C), we first assessed the impact on EEF1D expression. Consistent with our observations in KSHV-negative cells, KSHV reactivation by Dox treatment led to a significant downregulation of EEF1D mRNA and protein levels; however, this silencing effect was effectively reversed upon PATZ1 knockdown (Fig. 11A and B). These data confirm that PATZ1 is a critical mediator of RTA-induced EEF1D suppression during actual viral reactivation.

We further investigated whether the rescue of EEF1D expression resulted from the disruption of the epigenetic silencing machinery characterized in Fig. 10. BSP analysis confirmed that while viral reactivation induced robust promoter hypermethylation at the core regulatory hub, PATZ1 depletion almost entirely abolished this effect, maintaining the promoter in a predominantly hypomethylated state (Fig. 11C through F). This dramatic reduction in DNA methylation correlates with the recovery of *EEF1D* transcription, indicating that the recruitment of the RTA-DNMT3A complex to the chromatin is functionally dependent on PATZ1. Collectively, these findings demonstrate that PATZ1 serves as a critical adaptor that recruits the RTA-DNMT3A complex to the *EEF1D* promoter to enforce targeted epigenetic silencing during KSHV lytic reactivation.

## DISCUSSION

**Fig 12:**
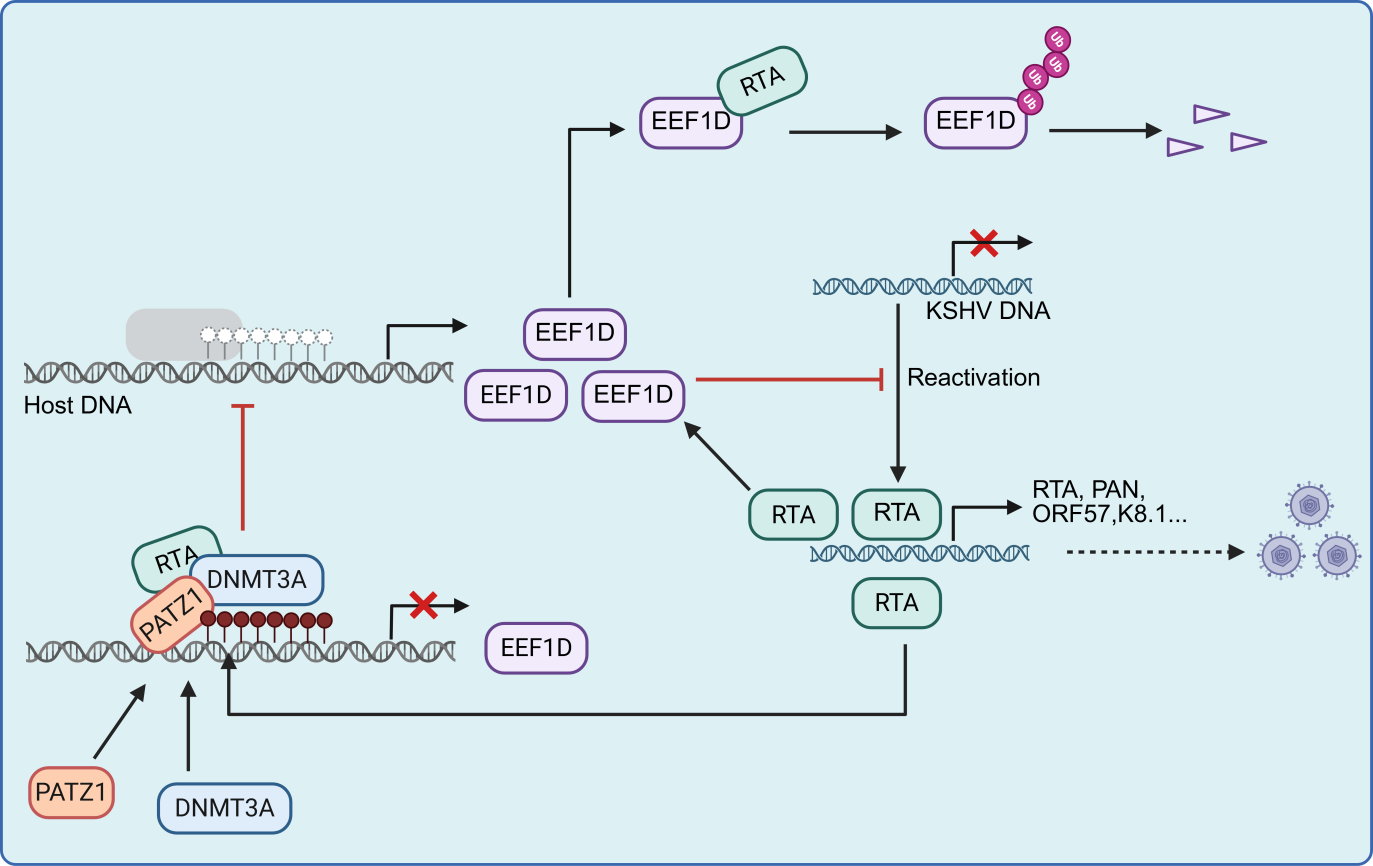
Model of RTA-mediated repression of EEF1D during KSHV lytic reactivation. EEF1D acts as a negative regulator of the KSHV reactivation. KSHV RTA counteracts this inhibition through two complementary mechanisms: (i) transcriptional repression, which serves as the primary mechanism, and (ii) direct interaction with the EEF1D protein, which promotes ubiquitin-proteasome-dependent degradation and serves as a secondary, supportive mechanism. Transcriptional repression is mediated by PATZ1, which facilitates RTA recruitment of DNMT3A to the *EEF1D* promoter, resulting in promoter hypermethylation and transcriptional silencing.

RTA is both a potent transcriptional activator and an E3 ubiquitin ligase that promotes the degradation of host restriction factors (65). Recent evidence suggests that RTA may modulate gene expression through additional, less well-defined mechanisms (38–40). Here, we show that EEF1D suppresses KSHV reactivation, whereas RTA counteracts this inhibition through two complementary mechanisms: ubiquitin-proteasome-mediated degradation and, more prominently, DNMT3A-dependent transcriptional repression. Mechanistically, the transcription factor PATZ1 directly associates with the *EEF1D* promoter and functions as a molecular scaffold that facilitates the recruitment of RTA and DNMT3A, leading to promoter hypermethylation and transcriptional silencing. Together, our results reveal an unanticipated role of RTA as a transcriptional repressor of host genes to enhance viral lytic reactivation (Fig. 12).

Our findings broaden the functional repertoire of the RTA. Traditionally characterized as a transcriptional activator, KSHV RTA was shown to function as a transcriptional repressor by inhibiting EEF1D transcription (Fig. 4 to 6). This role is consistent with the accumulating evidence that KSHV RTA exerts repressive activity on host genes. For example, it suppresses MDM2 expression by blocking Sp1- and p53-mediated transactivation (40), and Lingel et al. reported that RTA decreases MyD88 expression through RNA modulation (38). Furthermore, our comparative analysis revealed that the repressive effect of RTA on EEF1D is conserved in KSHV, RRV, and EBV but is absent in MHV68 (Fig. 6). This finding aligns with reports that EBV-RTA represses the transcription of *MYC*, *CCND1*, *JUN*, *IRF3*, and *IRF7* (41, 42) and that KSHV, but not MHV68, LANA induces pronounced DNA bending upon binding to terminally repeated viral DNA (66), suggesting that evolutionary divergence underlies these functional differences. Taken together, previous and current findings demonstrate that KSHV RTA functions not only as a transcriptional activator but also as a transcriptional repressor.

Understanding the transcriptional repression mechanisms of RTA is critical for elucidating how KSHV evades host antiviral responses; however, this area remains poorly explored. Lin and colleagues reported that KSHV RTA represses *MDM2* by inhibiting Sp1- and p53-mediated transactivation (40); however, no direct physical interaction was detected, leaving the underlying mechanism unresolved. Regarding epigenetic regulation, Chen et al. showed that EBV Rta induces promoter hypermethylation to disrupt CTCF binding and represses host gene transcription (42). However, in their study, the recruitment of DNMTs was attributed to an indirect effect of ROS because no direct Rta-DNMT interaction was observed. In contrast, our study defines the precise molecular machinery underlying this phenomenon. We found that KSHV RTA promotes hypermethylation of the *EEF1D* promoter specifically via DNMT3A, but not DNMT1 or DNMT3B (Fig. 8), indicating the selective engagement of the *de novo* methylation machinery. Notably, unlike EBV Rta, which directly binds to target promoters, KSHV RTA lacks canonical binding sites in the *EEF1D* promoter and instead relies on the transcription factor PATZ1 to recruit DNMT3A (Fig. 9 to 11). The essential role of PATZ1 supports a “transcription factor-guided recruitment” model, in which PATZ1 serves as a molecular bridge that enables RTA to orchestrate targeted promoter hypermethylation and transcriptional silencing. Given the broad range of host transcription factors exploited by herpesviruses, similar TF-RTA-DNMT3A complexes may operate at additional loci, highlighting the versatile strategy by which viral RTAs co-opt host cofactors for epigenetic regulation.

EEF1D functions as an inhibitor of KSHV reactivation, revealing a previously unrecognized antiviral role for this stress response regulator. Our data show that EEF1D suppresses KSHV lytic replication by reducing viral gene expression and progeny production. Notably, despite the RTA-dependent nuclear recruitment of EEF1D (Fig. 1D), this relocalization does not appear to impair general translational machinery. Specifically, EEF1D did not diminish RTA expression or its ability to activate lytic promoters and may modestly enhance RTA-dependent transcriptional activity (Fig. 4). Rather than translational interference, the observed partial (approximately 50%) yet biologically substantial suppression implies that EEF1D exerts a rate-limiting effect through indirect and saturable signaling networks. One key function of EEF1D is the induction of heme oxygenase 1 (HO1), which lowers ROS levels (47, 67, 68). Consistently, EEF1D overexpression has been reported to reduce ROS (49), while ROS, including H_2_O_2_, promote KSHV reactivation in latently infected cells (69). These observations support the hypothesis that EEF1D inhibits KSHV reactivation by upregulating HO1 and attenuating ROS accumulation. Further studies are needed to elucidate this pathway and determine whether additional stress-related functions of EEF1D contribute to its antiviral activity.

Finally, our results emphasize the broader theme of the viral manipulation of host epigenetic pathways. DNA methylation is a critical regulator of herpesvirus latency and reactivation. Analogous strategies include the KSHV LANA-mediated recruitment of DNMT3A to cellular promoters (70), EBNA3C-driven silencing of RASSF1A through DNMTs (71), and EBV RTA-mediated disruption of CTCF binding via methylation (42). Our findings extend this paradigm by demonstrating that KSHV RTA, a classical activator, also functions as a repressor through DNMT3A-dependent silencing.

In summary, this study identified EEF1D as a novel host factor that limits KSHV replication and demonstrated that RTA counteracts its antiviral activity through two complementary mechanisms: ubiquitin-proteasome-mediated degradation and, more prominently, PATZ1- and DNMT3A-mediated transcriptional silencing. These findings expand the functional repertoire of RTA, revealing its role as a transcriptional repressor, in addition to its established functions as a transcriptional activator and E3 ubiquitin ligase. By uncovering a mechanism by which a viral protein co-opts host transcription factors and epigenetic modifiers to silence antiviral genes, our study provides new insights into how KSHV coordinates its lytic and latent cycles. Future studies are important to explore the broader impact of RTA-mediated transcriptional repression on viral replication and pathogenesis and to determine whether similar regulatory strategies operate at other host loci.

## MATERIALS AND METHODS

### Cell culture

HEK293T, iSLK, iSLK.RGB, and the stably transduced iSLK.RGB-Flag-vector or iSLK.RGB-Flag-EEF1D cells were cultured in DMEM (Biological Industries, C3103-0500) supplemented with 10% fetal bovine serum (FBS; Biological Industries, C04001-500), 1% penicillin-streptomycin (Gibco, 15140122), and selective antibiotics: 1.5 μg/mL puromycin (Sigma-Aldrich, P8833), 0.5 mg/mL G418 (Sigma-Aldrich, A1720), 0.5 mg/mL hygromycin B (Roche, 10842555001), and 50 μg/mL blasticidin (Sigma-Aldrich, 15205). KSHV-positive B cell lymphoma lines (BCBL1, BCBL1-Flag-vector, and BCBL1-Flag-EEF1D) were maintained in RPMI 1640 (Biological Industries, C3010-0500) with 10% FBS. All cells were incubated at 37°C with 5% CO_2_. Lytic reactivation was induced by Dox (Sigma-Aldrich, 324385) in iSLK-derived cells and phorbol 12-myristate 13-acetate (TPA; MedChemExpress, HY-18739) in BCBL1-derived cells.

### Plasmids

The coding sequence of the long isoform of human EEF1D (NCBI RefSeq Isoform 1, NP_001123525.3) was amplified from iSLK.RGB cDNA and subcloned into Strep-Flag-pCDH (pCDH-SF), HA-pCMV (pCMV-HA), and pGEX-4T-1 vectors. Full-length DNMT1, DNMT3A, DNMT3B, PATZ1, CTCF, KLF5, and KLF15 were amplified from HEK293T cDNA and cloned into pCDH-SF. PATZ1 was additionally inserted into the Myc-pCMV vector. KSHV RTA expression constructs (pCDH-Flag-RTA, pCMV-HA-RTA, and pET-30a-RTA) were described previously (53, 72). Human and mouse *EEF1D* promoter fragments (bp −2095 to +395 relative to the transcription start site) were cloned into the pGL3-Enhancer vector for luciferase reporter assay. Primer sequences are listed in Table S1.

### Antibodies and reagents

Primary antibodies used included: anti-EEF1D (Proteintech, 10630-1-AP; note that the specific long isoform studied here [Isoform 1, 647 aa] migrates at ~95 kDa, distinct from the canonical short isoform), anti-RTA (an in-house rabbit polyclonal antibody generated and validated in our laboratory) (53, 73), anti-Flag (Sigma, F1804), anti-HA (Sigma, H6908), anti-Myc (ABclonal, AE070), anti-DNMT3A (ABclonal, A19659), anti-PATZ1 (Santa Cruz Biotechnology, sc-390577 X), anti-KLF5 (Proteintech, 21017-1-AP), anti-His (ABclonal, AE003), anti-GST (ABclonal, AE001), anti-SMC6 (Proteintech, 14465-1-AP), anti-α-Tubulin (Sigma, T5168), anti-UBC (ABclonal, A3207), rabbit control IgG (ABclonal, AC005), and mouse control IgG (ABclonal, AC011). Peroxidase-conjugated secondary antibodies were purchased from Jackson ImmunoResearch. Other reagents included Anti-Flag M2 affinity gel (Sigma-Aldrich, A2220-25ML), Pierce protein A agarose (Invitrogen, 20333), Pierce protein G agarose (Invitrogen, 20399), InvivoRNA (InvivoGene, IVG1101-10), Fugene HD Transfection Reagent (Promega, E2311), CHX (MedChemExpress, HY-12320), MG132 (MedChemExpress, HY-13259), 5-Aza-2’-deoxycytidine (5-AZA-CdR; MedChemExpress, HY-A0004), protease inhibitor cocktail (Sigma, P8340), and PMSF (Beyotime, ST506).

### Co-immunoprecipitation and immunoblotting

Cells were lysed on ice in IP buffer containing protease inhibitors. Lysates were cleared and incubated with anti-Flag M2 affinity gel or the corresponding antibodies with protein A/G beads overnight at 4°C. The immunocomplexes were washed and boiled in SDS loading buffer. Proteins were resolved by SDS-PAGE, transferred to nitrocellulose membranes, blocked, and probed with specific primary and secondary antibodies.

### Glutathione S-transferase (GST) pulldown assay

His-RTA and GST-EEF1D fusion proteins were expressed in *Escherichia coli* BL21 (DE3, Tsingke, TSC-E01) cells. Bacterial pellets were lysed by sonication, and GST or GST-EEF1D was purified using a GST-tag Protein Purification Kit (Beyotime, P2262). His-RTA was purified using a His-tag Protein Purification Kit (Beyotime, P2229S). For pulldown assays, purified GST or GST-EEF1D was incubated with anti-GST immunomagnetic beads (Biolinkedin, L1014A) for 4 h at 4°C, washed, and mixed with purified His-RTA overnight. The beads were extensively washed, and the bound proteins were eluted and analyzed using immunoblotting.

### RNA isolation and quantitative PCR

Total RNA was extracted using the PaPure RNA Kit (Magen, R4011-03) and reverse-transcribed into cDNA using HiScript III RT SuperMix (Vazyme, R323-01). Real-time qPCR was performed using ChamQ SYBR qPCR Master Mix (Vazyme, Q311-02/03) on a QuantStudio 6 Flex System (Thermo Fisher). Each sample was analyzed in at least three technical replicates. Data presented in the figures are from a representative experiment and are expressed as the mean ± standard deviation (SD) of the technical replicates. Similar results were obtained in at least three independent biological experiments for each experiment. Relative gene expression was calculated using the 2^-∆∆Ct^ method, with *GAPDH* as the internal normalization control. Statistical significance was determined using an unpaired Student’s *t*-test. Differences were considered statistically significant at *P* < 0.05. Primer sequences are listed in Table S1.

### RNA interference

RNA targeting *EEF1D*, *RTA*, *DNMT1*, *DNMT3A*, *DNMT3B*, *PATZ1*, and *KLF5* (GenePharma) was transfected using InvivoRNA reagent. Knockdown efficiency was assessed 48 h later using qRT-PCR and immunoblotting. The siRNA sequences are provided in Table S1.

### Dual-luciferase reporter assay

HEK293T cells were seeded in 12-well plates and cultured until they reached approximately 70% confluence. Cells were then co-transfected using PEI with 0.5 μg of the specific firefly luciferase reporter plasmid, 0.5 μg of the effector plasmid (Flag-vector or Flag-RTA), and 15 ng of pRL-TK (Renilla luciferase) plasmid as an internal control. After 48 h, the cells were lysed, and firefly and Renilla luciferase activities were measured using the Dual-Luciferase Reporter Assay System (Promega, E1960) according to the manufacturer’s instructions. To account for variations in transfection efficiency, firefly luciferase activity was normalized to Renilla luciferase activity, and the data are presented as the relative ratio (Fluc/Rluc).

### BSP

Genomic DNA was extracted (Tiangen, A1125A), and bisulfite conversion was performed using the EpiArt DNA Methylation Bisulfite Kit (Vazyme, EM101-01). The CpG-rich regions of the *EEF1D* promoter were predicted using MethPrimer. Amplified fragments were cloned into the pCE2 TA/Blunt-Zero vector (Vazyme, C601-01), transformed into *E. coli* DH5α, and positive clones were sequenced to assess their methylation status.

### ChIP

ChIP assays were performed using the BeyoChIP Enzymatic ChIP Assay Kit (Beyotime, P2083S). Immunoprecipitated DNA was analyzed by qRT-PCR, and enrichment was calculated relative to the IgG controls. Primer sequences are provided in Table S1.

### Stable cell lines

Lentiviruses were generated in HEK293T cells by cotransfecting Δ8.9, pVSV-G, and either pCDH-Flag-EEF1D or an empty vector. Viral supernatants collected at 72 h were used to infect iSLK.RGB or BCBL1 cells by spinoculation at 2,500 × *g* for 2 h at 37°C. Infected cells were selected with 50 µg/mL blasticidin, and stable expression was verified by qRT-PCR and immunoblotting using a specific anti-EEF1D antibody. It should be noted that the high overexpression efficiency achieved (>100-fold) necessitated short exposure times to avoid signal saturation, which may render the relatively low endogenous levels of the EEF1D protein (approximately 95 kDa) in the vector control below the visual threshold.

### Viral DNA quantification

iSLK.RGB (2–4 × 10^5^ cells) or BCBL-1 (1–2 × 10^6^ cells) with overexpression or knockdown of EEF1D were cultured in six-well plates and induced with Dox or TPA for 72 h. For extracellular viral DNA quantification, 200 μL of the cell culture supernatant was collected. For intracellular viral genomic DNA, the remaining cells were harvested and dissociated using trypsinization. The total cell number (*N*) for each sample was determined by manual counting using a hemocytometer. To ensure accuracy, the samples were normalized based on the actual cell count prior to DNA extraction, ensuring that equal numbers of cells were subjected to lysis.

DNA was extracted from both the supernatant and cell pellets using the TIANamp Genomic DNA Kit (Tiangen, DP304), following the manufacturer’s instructions. Absolute viral copy numbers were determined by qPCR using a standard curve generated from K9 plasmids (73). For intracellular viral genomic DNA, due to the high copy number exceeding the linear detection range of the qPCR instrument, the template DNA was diluted 50-fold prior to amplification.

The final viral copy numbers were calculated using the raw absolute quantification value (*Q*, copies per reaction) derived from a standard curve. The intracellular viral load was expressed as copies per cell, and the extracellular viral load was expressed as copies per mL, calculated using the following formulas:


$$\begin{array}{rl} & \text{ Intracellular }(\text{ Copies }/\text{ cell })=\frac{Q\times (200/4)\times 50}{N}\\ & \text{ Extracellular }(\text{ Copies }/mL)=Q\times (200/4)\times 5 \end{array}$$

where *Q* is the raw copy number per reaction obtained from the qPCR, 200 is the elution volume (μL) used for DNA extraction, 4 is the volume of DNA template (μL) added to each, 50 is the dilution factor for intracellular DNA templates, *N* is the total number of cells used for DNA extraction, and 5 is the conversion factor from 200 μL aliquot of supernatant to 1 mL aliquot.

### Infection of HEK293T cells with progeny virus

One day before infection, HEK293T cells were seeded into 12-well plates at an appropriate density. The culture medium was removed and replaced with 2 mL of cell culture supernatant collected 96 h post-induction. To enhance viral attachment and infectivity, spinoculation was performed by centrifuging the plates at 2,500 × *g* for 2 h at 37°C. After centrifugation, the supernatant was replaced with fresh complete medium.

At 24 h post-infection, viral infectivity was assessed by detecting RFP signals using fluorescence microscopy. For quantification, three random fields of view were captured for each sample. The images were processed using Cellpose (74) for automated cell segmentation to define cellular boundaries, and the number of RFP-positive cells was counted using ImageJ software (75).

### Statistical analysis

All statistical analyses were performed using GraphPad Prism. Data are shown as mean ± SD from at least three independent experiments. Comparisons were made using unpaired Student’s *t*-tests. ns, not significant; *, *P* < 0.05; **, *P* < 0.01; ***, *P* < 0.001; ****, *P* < 0.0001.

## Data Availability

All data generated or analyzed during this study are included in this published article and its supplemental material.

## SUPPLEMENTAL MATERIAL

Supplemental material jvi.01793-25-s0001.pdf Figure S1; Tables S1 and S2.

ASM does not own the copyrights to Supplemental Material that may be linked to, or accessed through, an article. The authors have granted ASM a non-exclusive, world-wide license to publish the Supplemental Material files. Please contact the corresponding author directly for reuse.
