# Supplementary material for: Repression of EEF1D by KSHV RTA promotes viral lytic reactivation
Source: J Virol. 2026 Mar 10;100(4):e01793-25. doi: 10.1128/jvi.01793-25 (PMC13098232; doi:10.1128/jvi.01793-25)
Supplement: Supplemental material — Figure S1; Tables S1 and S2. [file jvi.01793-25-s0001.pdf]

Supplementary Figure

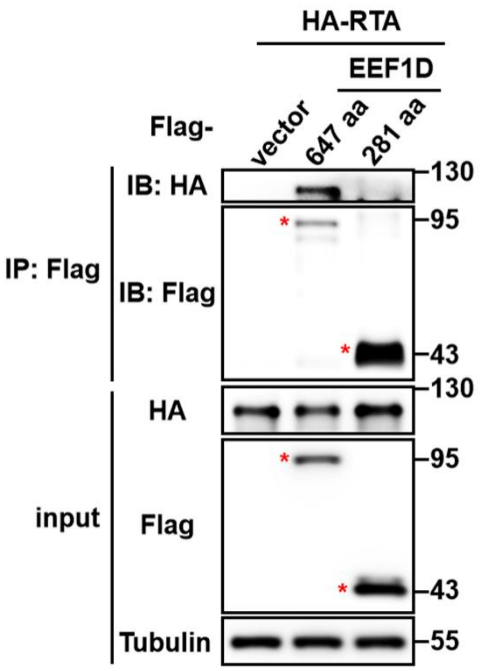

Fig. S1 Isoform-specific interaction between RTA and EEF1D. HEK293T cells were co-transfected with plasmids encoding HA-RTA and either Flag-vector, Flag-EEF1D long isoform (647 aa), or Flag-EEF1D short isoform (281 aa). Cell lysates were immunoprecipitated (IP) with anti-Flag M2 affinity gel, followed by immunoblotting (IB) with anti-HA and anti-Flag antibodies to detect the interaction. Whole cell lysates (Input) were analyzed to verify the expression levels of HA-RTA and Flag-EEF1D isoforms. Tubulin served as a loading control. Red asterisks indicate the migration positions of the long (~95 kDa) and short (~43 kDa) Flag-EEF1D isoforms.

Supplementary Tables

Table S1 Sequences used in our study

Sequence name	Sequence of oligonucleotide (5'-3')
**PCR amplification**	
PCDH-Flag-EEF1D-F	TACCGGACTCAGATCTCGAGATGAGGAGCGGGAAGGCC
PCDH-Flag-EEF1D-R	GGTACCGTCGAGTGCAGAATTCCAGATCTTGTTGAAAGCTGCGATATCGACA
PCDH-Flag-DNMT1-F	TACCGGACTCAGATCTCGAGATGCCGGCGCGTACCGCC
PCDH-Flag-DNMT1-R	GGTACCGTCGAGTGCAGAATTCCAGTCCTTAGCAGCTTCCTCCTCCTTTATTTTAGCTG
PCDH-Flag-DNMT3A-F	TACCGGACTCAGATCTCGAGATGATGCCCGCCATGCCCTCCAGCGG
PCDH-Flag-DNMT3A-R	GGTACCGTCGAGTGCAGAATTCCATTACACACACGCAAAATACTCCTTCAGCGGAGCG
PCDH-Flag-DNMT3B-F	TACCGGACTCAGATCTCGAGATGAAGGGAGACACCAGGCAT
PCDH-Flag-DNMT3B-R	GGTACCGTCGAGTGCAGAATTCCATTCACATGCAAAGTAGTCCTTCAGAGG
PCDH-Flag-PATZ1-F	TACCGGACTCAGATCTCGAGATGGAGCGGGTGAACGACG
PCDH-Flag-PATZ1-R	GGTACCGTCGAGTGCAGAATTCCAGCCAACAGGCCACTGGGTAA
PCDH-Flag-CTCF-F	TACCGGACTCAGATCTCGAGATGGCCTTTGTGACCAGTGGAG
PCDH-Flag-CTCF-R	GGTACCGTCGAGTGCAGAATTCCACCGGTCCATCATGCTGAGGAT
PCDH-Flag-KLF5-F	TACCGGACTCAGATCTCGAGATGGCTACAAGGGTGCTGAGC
PCDH-Flag-KLF5-R	GGTACCGTCGAGTGCAGAATTCCAGTTCTGGTGCCTCTTCATATGCAGG
PCDH-Flag-KLF15-F	TACCGGACTCAGATCTCGAGATGGTGGACCACTTACTTCCAGTG
PCDH-Flag-KLF15-R	GGTACCGTCGAGTGCAGAATTCCAGTTCACGGAGCGCACGGA
PCMV-HA-EEF1D-F	ATGGCCATGGAGGCCCGAATTCCAATGAGGAGCGGGAAGGCC
PCMV-HA-EEF1D-R	ATGTCTGGATCCCCGCGGCCGCTCAGATCTTGTTGAAAGCTGCGATATCGAC
pGEX-4T-1-EEF1D-F	ATCTGGTTCCGCGTGGATCCATGAGGAGCGGGAAGGCC
pGEX-4T-1-EEF1D-R	TCACGATGCGGCCGCTCGAGTTAGATCTTGTTGAAAGCTGCGATATCGAC
PCMC-C-MYC -PATZ1-F	GCTCTAGCCCGGGCGGATCCATGGAGCGGGTGAACGACG
PCMC-C-MYC -PATZ1-R	TCGACGGTATCGATAAGCTTGCCAACAGGCCACTGGGTAA
PGL3-Enhancer-EEF1D-F	ACATTTCTCTATCGATAGGTACCTCCAGGTGCAGCCACTCCGCAAC
PGL3-Enhancer-EEF1D-R	CTTAGATCGCAGATCTCGAGCCACGGAGACGGCAGCAAACACA
PGL3-Enhancer-mEEF1D-F	ACATTTCTCTATCGATAGGTACCAGAAGAGCACTCGAACTGCACCA
PGL3-Enhancer-mEEF1D-R	CTTAGATCGCAGATCTCGAGATCTCCCAACACGCCGAACCC
**qPCR**	

EEF1D-F	CCTCCCTGTCTCACCGACCC
EEF1D-R	CAGGCCCGTTCATCTGCTCGT
RTA-F	AGACCCGGCGTTTATTAGTACGT
RTA-R	CAGTAATCACGGCCCCTTGA
PAN-F	TTTCGGCTAGGTTTTCCGTCC
PAN-R	ATCCAATGCAATAACCCGCAAG
ORF57-F	TGGACATTATGAAGGGCATCCTA
ORF57-R	CGGGTTCGGACAATTGCT
K8.1-F	AAAGCGTCCAGGCCACCACAGA
K8.1-R	GGCAGAAAATGGCACACGGTTAC
ORF73-F	GACTTCTGGATCCCACGCTA
ORF73-R	CATGTGCCCAGTAACTTACGC
GAPDH-F	CTCTGCTCCTCCTGTTCGAC
GAPDH-R	GCGCCCAATACGACCAAATC
DNMT1-F	ACCTGGCTAAAGTCAAATCCCTT
DNMT1-R	ATTCACTTCCCGGTTGTAAGCA
DNMT3A-F	GGCAAAGACCAGCATTTTCCTGT
DNMT3A-R	ACTGGCACGCTCCATGACC
DNMT3B-F	CGACAGGCTACCTAGTCCTCA
DNMT3B-R	ATTTGATATTCCCCTCGTGCTT
PATZ1-F	TACACTTCCCGCATCGTGGT
PATZ1-R	ATGACTTCCTGGCAGATCTCG
KLF5-F	ATCCTATGCTGCTACAATTGCT
KLF5-R	TCGTTTCTCCAAATCGGGGTT
K9-F	GTCTCTGCGCCATTCAAAAC
K9-R	CCGGACACGACAACTAAGAA
**siRNA**	
siEEF1D#1	GCCGCAACAUCUUAGGGAATT
siEEF1D#2	CGGGUGGCCUGGUGCCUCGTT
siRTA#1	GCCAGUUUGUCAUUAGCAAAC
siRTA#2	GCAAUUUCAACCACCGCAAUG
siDNMT1#1	CAGUCCCGAGUAUGCGCCCAUAUUU
siDNMT1#2	CAGCACAAACUGACCUGCUUCAGUG
siDNMT3A#1	GAGCAUGGCAGGAUAGCCAAGUUCA
siDNMT3A#2	GAGGACAUCUUAUGGUGCACUGAAA
siDNMT3B#1	CGCCUCAAGACAAAUUGCUAUAACA
siDNMT3B#2	GGUAGGAAAGUACGUCGCUUCUGAA
siPATZ1#1	CAUGCGGUCUAUGUGGUAAGGUGUU
siPATZ1#2	UGCACACUAUCAGCUCCAAGGUAUU
siKLF5#1	CAAUUGCUUCUAAACUGGCAAUUCA
siKLF5#2	CCGGAUCUAGAUAUGCCCAGUUCUA

**ChIP-qPCR**	
rs57-F	GTTGCCGCTGCTGGACGTT
rs57-R	TGGCGTGAACCCCGGGGGA
HDX-F	GCTCCTGAATCATCCATAA
HDX-R	CAATTATCAACTTAGAGACACAT
Region A-F	CGCAGGACACAGCCAGTCG
Region A-R	CGGGTGCTGTTCCTGTCCAA
Region B-F	ACGCCTGGGACAAATTCCTCA
Region B-R	CGCCACCACAACCAGGACA
Region C-F	GCCAGCAGCACCGTTACGC
Region C-R	CGTCACCGGCCTCTACTGGA
Region D-F	AGCTTCCAGTAGAGGCCGGTGAC
Region D-R	GAGCCCGCGCTGCCCTCCG
**Bisulfite sequencing PCR (BSP)**	
EEF1D-F	TATATTGGTAGTTGGGTTTGGGTAG
EEF1D-R	CCACCACAACCAAAACAAATTCC

Table S2 Candidate transcription factors predicted to target EEF1D promoter

HumanTFDB	GTRD	UCSC-JASPAR (minimore score 600)
SMAD1	IKZF3	Nrf1
BRD4	APC	PATZ1
EP300	SAP130	KLF15
SPI1	SMARCB1	KLF5
MYB	BRCA2	KLF16
TCF4	CEBPB	ZBTB1
SP2	SP7	CTCF
SRF	SMAD3	
REST	INTS3	
BMI1	ESR2	
FOXA2	MSC	
KLF5	ME1	
SMARCA4	BPTF	
SP3	CXXC4	
JUND	PRDM11	
SP1	PRDM10	
SP4	PRDM9	
ORC1	FXR1	
FOXG1	FXR2	
E2F1	RAD21	
POU5F1	ZEB1	
MYC	MGA	
BTAF1	PEX2	
SRC	SIRT1	
CDK9	PIAS1	
HDAC2	AGO2	
NOTCH1	TBX5	
BRD3	TEAD3	
KMT2A	ZBTB18	
CDK7	ZBTB2	
CASP8AP2	PBX3	
RELA	PTBP1	
IRF1	PBX1	
SMAD4	MNT	
ZBTB33	NFIA	
ZBTB7A	NFKB1	
ZBTB7B	DTL	
BRCA1	STAT2	
USF1	NFYC	
BHLHE40	CBFB	

YY1	RUNX2	
ELL2	AGO1	
CBFB	LEO1	
POLR2A	TBX21	
EGR1	RING1	
SUZ12	KLF17	
CDK8	ZNF48	
MAZ	BRD4	
KLF9	T-Cell	
RUNX1	RBL2	
ELK3	BHLHE40	
ZNF143	CREM	
CHD1	HDAC2	
SUMO2	EHF	
MED1	DDX21	
GABPA	ZBTB7A	
IRF3	RBPJ	
MAX	OR2M7	
MXI1	UBTF	
NFYA	LCORL	
NFYB	INTS12	
PBX3	EZH2	
SREBF1	SOX13	
TBP	TP53BP1	
USF2	ZFHX3	
ZFP42	MEIS2	
CREB1	ZBTB6	
E2F4	GTF2A2	
ELF1	ETS2	
NCOR1	ASH1L	
NRF1	BRCA1	
HSF1	ZNF778	
CTCF	EHMT2	
OGT	TFAP2C	
TBL1XR1	SQSTM1	
ZNF92	IKZF2	
RFX2	THRB	
MYH11	ZNF629	
PHF8	CHD4	
TAF1	ZNF740	
ZNF384	CHD8	
UBTF	NFKBIZ	
VEZF1	ZBTB26	

GTF2B	GABPA	
PBX1	GABPB1	
PRAME	FOXA2	
RB1	NR3C1	
PLAG1	KDM5B	
THAP1	TEAD1	
SUMO2/SUMO3	BRD9	
CLOCK	RCOR1	
NPAT	ZNF574	
FOS	KDM7A	
MZF1	LARP7	
KDM1A	CASP8AP2	
PRKDC	FOSL2	
RXRA	PGM3	
ERG	IRF2	
LYL1	KLF15	
EZH1	TCF4	
BARX2	GATAD1	
STAT5B	SUZ12	
LMNB1	NFYB	
POU2F1	ZNF701	
HOXA1	TFDP1	
PAX5	ZC3H8	
RBCK1	ZBTB33	
TRIM24	ARNTL	
EGR4	NSD2	
AP2	EP300	
GLI2	ZNF687	
TAF3	OVOL2	
ZFX	RBFOX2	
NCOR2	ETV2	
ETS1	ZNF83	
MAFB	NFKB2	
TCF7L2	E4F1	
PCGF2	SOX8	
NR3C2	ZNF449	
KAT5	SETDB1	
TFAP2B	SFPQ	
KLF4	BRD2	
PLAGL1	E2F8	
STAG1	SMARCA2	
STAT1	CRTC2	
NR3C1	CTNNB1	

GTF3C2	CTCFL	
PPARGC1A	SMARCA4	
JMJD6	PCGF2	
KDM5B	BMI1	
STAT2	ZNF263	
TBL1X	SIN3A	
MTF1	CBFA2T3	
SP1:SP3	PTEN	
ESR1	GTF2B	
ZNF281	RBBP5	
PURA	ZNF711	
EZH2	BRD3	
E2F7	KLF4	
NR5A2	MAFB	
ARRB1	YY1	
STAT3	CDK7	
CTCFL	ZNF639	
HDAC6	KLF11	
RXRG	ELK3	
PATZ1	ZMYM2	
CNOT3	c-myc	
SOX2	JUN	
RAD21	CENPA	
KAT2B	p65	
NR2C2	DUX4	
TCF12	PPARG	
GTF2I	SMARCC1	
ARNT	ZNF770	
ASCL1	KMT2B	
WT1	ZFP42	
GMEB2	DDX5	
SUMO1	XRCC5	
TFAP2D	HSF1	
PPARD/PPARG	ZNF395	
CTBP2	ZNF614	
GATA1	GATA6	
TFAP2A	GTF2F1	
FOXP1	XBP1	
ATF3	MITF	
ARID3A	NEUROG2	
PPARG:RXRA	ZNF610	
HIC1	UPF1	
PPARG	DEK	

IRF5	SRSF7	
ASCL2	KLF10	
ARNT2	BCL11A	
EBF1	MYRF	
SMC3	SP1	
SMAD3	TAF3	
KLF1	MYNN	
RNF2	CTBP1	
TRIM28	REST	
TFAP2C	NCOA1	
ZC3H8	TAF7	
STAT5A	ZBTB42	
EGR2	IRF4	
RARG	INO80	
NFE2	NCOR1	
ZBTB17	RARG	
SMC1A	ZSCAN30	
TFCP2	SP3	
LMO2	REL	
TAL1	SP4	
GLIS3	NOTCH1	
E2F3	SMAD2	
MRE11A	SUMO2	
HEY2	NR1H3	
TCF3	ETV4	
REPIN1	HES1	
LRH1	NFIL3	
ZNF148	DDIT3	
MBD2	SMAD1	
GATA4	FUS	
MYOD1	SKIL	
NHLH1	STAG1	
MAFA	HIRA	
TP73	PHF8	
E2F6	NCOA3	
EGLN2	HOXC8	
KLF11	ZNF217	
CTNNB1	ZNF121	
ZNF76	MIXL1	
NEUROD1	CDKN1B	
LRF	TEAD4	
ZNF263	USP7	
ZNF219	WDR5	

C17orf96	ZFHX2	
NFIC	TARDBP	
HCFC1	ZMYND11	
AR	AHR	
BRD7	NRF1	
TOP1	ERCC2	
RBL2	NR1H2	
ZNF711	NR2F6	
KDM4A	NR2F1	
SMAD2/SMAD3	PADI2	
KLF6	SREBF1	
FOXA1	FOXH1	
TP63	ZNF335	
FOXM1	ZNF280D	
SMAD2	ZNF554	
PAX3	DMAP1	
EED	HIF1A	
ZNF589	HNRNPK	
KLF15	DNMT3A	
TP53	EN1	
RREB1	NFE2L1	
PGR	ELF4	
AP4	RBM39	
NR2F6	TRIM25	
BCL6	ATRX	
NFE4	KDM6B	
NFYC	FOXO3	
FOSL1	ZNF35	
MLXIPL	NCOA2	
SIN3A	GRHL3	
HES1	ZBTB5	
ICE2	MBL2	
CDX2	BRPF3	
NR5A1	SUPT6H	
SF1	FLI1	
E2F2	HNF1B	
ETV1	RBM14	
ATF1	CBX3	
TCF21	LMNA	
ATF2	INTS13	
SIX5	EBP	
BARHL1	PCBP2	
POLR3A	ZFP64	

RARA	PCBP1	
ZBTB14	SRSF4	
RBPJ	RBBP4	
NR6A1	ESRRA	
LIN9	VDR	
DEAF1	KLF3	
EWSR1	RUNX1	
THAP11	ONECUT2	
HIF1A	EPAS1	
RUNX2	ELF3	
HESX1	PHF6	
TLX1	EP400	
ESRRA	KDM5A	
NKX2-1	OLIG2	
CIITA	EMSY	
CACD	NR4A1	
ETS2	MTOR	
TTF1	KDM3A	
GATA2	THAP11	
ELK1	HMGXB4	
ELK4	ARID4B	
TFDP1	RUNX3	
KLF8	EED	
EOMES	ZSCAN16	
ZBTB18	YAP1	
FOXK1	GRHL2	
ESR2	ZNF341	
VDR:CAR:PXR	EBF1	
LEF1	EGR3	
RFX5	BAP1	
RCOR1	BCHE	
NFATC1	DAXX	
TEAD4	ARID5B	
VDR	CBX1	
NKX2-8	RXRA	
IKZF1	PML	
PPARA	GATAD2B	
CREBBP	HNF4A	
TFAP4	CREBBP	
GFI1B	HDGFL2	
PPAR:HNF4:COUP:RAR	SETX	
HOXA9	FIP1L1	
ELF2	MBD3	

GLYR1	ZNF280A	
OTX2	HDAC1	
KAT8	ELF1	
MYOG	HOMEZ	
EPAS1	SRF	
WHSC1	RELA	
RP58	MLX	
LEF1:TCF1	SRSF1	
ZNF250	DPF2	
HNF4G	KDM5C	
MYCN	APOBEC3B	
KDM5A	RFXANK	
MECP2	MLLT1	
NANOG	MECOM	
TEAD2	HMG20A	
SREBF2	FOXK2	
1-Dec	ZXDC	
FLI1	FOXK1	
TEAD1	ZNF320	
HNF4A	LMO1	
ZBTB4	NEUROD1	
ATF4	CC2D1A	
NR2F2	ADA2	
NR0B1	IRF3	
HDAC1	SSU72	
INSM1	ETS1	
TLX1:NFIC	LYL1	
CTF1	ZBTB16	
THRA	TET2	
ZNF217	HNRNPUL1	
BCL3	RAD51	
ETV3	EBF3	
ETV7	HINFP	
HAND1	HOXA4	
MAFK	ZNF600	
GRHL3	GATA4	
GATA3	BICRA	
CENPA	MLXIP	
ZIC3	NKX2-1	
SNAI1	ZBTB17	
STAT3:STAT3	GLIS3	
XBP1	RAG2	
EHF	FOXP1	

HIVEP2	TCF7L1	
GLI3	NFE2	
ESRRG	SS18	
ETV4	KLF1	
CEBPA	TAF1	
ERF	SMC1A	
JUN	IRF1	
RAC3	ATOH1	
CXXC1	ZFP1	
MYF6	MBD2	
TBX3	SUPT5H	
ETV5	RERE	
SAP30	CREB1	
NR2F1	CLOCK	
ZNF350	ZNF205	
SOX17	DNMT3B	
GLI1	OSR2	
STAT4	OGG1	
GTF2IRD1	AHRR	
ATF6	MAFF	
LXR:PXR:CAR:COUP:RAR	MIER3	
T	KLF13	
ZKSCAN1	TFAP2A	
ELF3	BCLAF1	
PIAS1	NCAPH2	
BHLHE41	NFATC3	
ZBED6	FOXD2	
BACH2	NELFE	
OVOL1	LEF1	
PLAU	NR2C2	
PPARD	ESR1	
RUNX3	PGR	
ETV2	ZSCAN9	
HNF4:COUP	RB1	
TAL1:TCF3	KAT2B	
SPDEF	HOXB13	
BCOR	ZNF350	
NR4A2	KDM6A	
NRIP1	JUND	
SPIB	DRAP1	
CEBPB	ATF7	
KLF13	TAL1	
CREM	LMNB1	

SETDB1	PATZ1	
FEV	HAND2	
PML	DACH1	
CBX3	TFE3	
PAX6	GTF3C2	
MIF1	SCRT2	
RXR:RAR_DR5	ETV6	
ELF4	MYOG	
RXR:RAR	MYOD1	
ZNF280D	CAT	
DDX5	SRSF3	
ZNF274	ELL2	
NR4A1	RUNX1T1	
NF1	MBD4	
NFE2L2	NR5A2	
NR1H4	NFIC	
SOX9	CUL4A	
REX1	RORC	
ZBTB6	KAT7	
CBX1	SP2	
ESRRB	ESCO2	
BPTF	MAX	
TCF7	TBL1XR1	
SALL4	CHD2	
TGIF1	SNRNP70	
GCM1	CHD1	
TTF2	SREBF2	
THRB	FOXO1	
IRF4	STAT5B	
GRHL2	KMT2C	
HAND2	MEN1	
ZIC1	NIPBL	
HINFP	KMT2A	
BRCA1:USF2	ARID2	
SMAD2:SMAD3:SMAD4	BCL3	
SOAT1	RNF2	
MTA3	INTS11	
NKX3-1	ZNF580	
RFX3	TP53	
NR2E3	TRPS1	
E2F8	SETD7	
HEY1	MXI1	
MAF	SPI1	

GMEB1	LDB1	
BCL11A	NFE2L2	
HSF	BCL11B	
ERMAP	ZHX2	
ENO1	HNRNPC	
	MORC2	
	ETV1	
	ERF	
	PRMT1	
	PAX2	
	MYC	
	SOX9	
	IVNS1ABP	
	PRPF4	
	FOS	
	HNRNPLL	
	METTL3	
	ZNF467	
	SOX2	
	ASCL1	
	TOP1	
	POU5F1	
	RBM25	
	CDX2	
	E2F4	
	ZNF143	
	KLF6	
	MED12	
	EGR2	
	ZSCAN22	
	ZBTB48	
	GLI2	
	POU2F2	
	NR2F2	
	SUPT20H	
	ARID3A	
	AFF1	
	NANOG	
	SALL3	
	ILF3	
	GLIS2	
	AFF4	
	GFI1	

	CDK8	
	ZBTB25	
	PALB2	
	STAT3	
	PRDM14	
	PLAG1	
	TAF15	
	SMARCE1	
	CNOT3	
	ATF2	
	SIN3B	
	ATF1	
	ATF3	
	E2F7	
	CDK9	
	KDM4A	
	RBM22	
	BRD7	
	MECP2	
	DCP1A	
	IRF9	
	TP63	
	ARNT	
	SIRT6	
	PDX1	
	HCFC1	
	PHF2	
	HIF3A	
	BCOR	
	NUP98	
	DMC1	
	PHF5A	
	GMEB2	
	TCF3	
	CBFA2T2	
	ZNF549	
	ASCL2	
	SMAD4	
	NFKBIA	
	NFIB	
	SAFB	
	TCF21	
	E2F3	

	KDM3B	
	MYB	
	MYBL2	
	CDK12	
	MXD4	
	BATF	
	PCF11	
	KDM2B	
	TWIST1	
	SCML2	
	ERG	
	MXD3	
	CRY1	
	OTX2	
	CPSF3	
	ETV7	
	ZNF423	
	GATA2	
	ZNF8	
	ZBTB20	
	TRIM28	
	MYH11	
	MAPK14	
	VEZF1	
	H2AFZ	
	NCOR2	
	SP5	
	PAX5	
	TLE3	
	GATA3	
	MAZ	
	TRIM24	
	ZNF324	
	HDGFL3	
	E2F6	
	AR	
	RARA	
	ID3	
	RELB	
	GLIS1	
	ME3	
	KDM4B	
	HEY1	

	GATA1	
	ZNF792	
	TCF7L2	
	SMAD5	
	AEBP2	
	ZNF382	
	NONO	
	SMARCC2	
	SAP30	
	TCF7	
	MAF	
	TFAP4	
	CTCF	
	FANCD2	
	CEBPD	
	ZIC5	
	CEBPA	
	FOXM1	
	ARNT2	
	SNAI2	
	HNRNPL	
	FGFR1	
	ZNF282	
	GFI1B	
	MBTD1	
	MCM7	
	E2F1	
	ZNF24	
	STAT1	
	ZNF3	
	JUNB	
	SUMO1	
	PHF20	
	STAT5A	
	STAT6	
	KLF9	
	KLF8	
	HEXIM1	
	U2AF1	
	KLF5	
	KDM4C	
	FOXA3	
	FOXA1	

	KAT8	
	TCF12	
	SSRP1	
	APP	
	ASH2L	
	ORC2	
	JDP2	
	ORC1	
	MTA3	
	CCAR2	
	ZFX	
	HDAC3	
	L3MBTL2	
	PARP1	
	KLF16	
	YY2	
	IKZF1	
	XRCC4	
	ZNF667	
	NELFA	
	GUCY1B3	
	ZBTB14	
	DOT1L	
	CSNK2A1	
	ZNF18	
	MYCN	
	HDAC6	
	STAT4	
	USF2	
	BARX1	
	HIC1	
	JMJD6	
	BCL6	
	HEYL	
	CEBPG	
	USF1	
	ERCC6	
	CBX8	
	NFATC1	
	MED26	
	ZBTB40	
	CCND2	
	SMC3	

	NFYA	
	ZIC2	
	PMEPA1	
	HNF4G	
	SUPT16H	
	HMGB1	
	ARID1B	
	ETV5	
	EGR1	
	SOX6	
	SOX5	
	ZNF444	
	ATF7IP	
	KDM1A	
	NFRKB	
	CCNT2	
	MUC22	
	CXXC1	
	GATAD2A	
	TBP	
	ZGPAT	
	SOX4	
	SIX5	
	ARID1A	
	HMGN3	
